# Eemian palaeogenetics demonstrates loss of diversity in modern fallow deer (*Dama dama*)

**DOI:** 10.1016/j.isci.2026.116204

**Published:** 2026-06-03

**Authors:** Alberto Rocha-Méndez, Patrick Arnold, Lutz Kindler, Sabine Gaudzinski-Windheuser, Wil Roebroeks, Fulco Scherjon, Michael Hofreiter

**Affiliations:** 1 Evolutionary Adaptive Genomics, Institute of Biochemistry and Biology, University of Potsdam, Karl-Liebknecht-Strasse 24-25, 14476 Potsdam, Germany; 2 Leibniz Zentrum für Archäologie - Neuwied Location, CC Pleistocene and Early Holocene Archaeology, MONREPOS Archaeological Research Centre and Museum for Human Behavioral Evolution, 56567 Neuwied, Germany; 3 Institute of Ancient Studies, Pre- and Protohistoric Archaeology, Johannes Gutenberg-University Mainz, 55116 Mainz, Germany; 4 Faculty of Archaeology, Leiden University, P. O. Box 9514, 2300 RA Leiden, the Netherlands

**Keywords:** Zoology, Phylogenetics, Paleobiology, Paleogenetics

## Abstract

The European fallow deer (*Dama dama*) is a widely distributed cervid that has experienced extensive human management since the Neolithic. Although several studies have examined relationships among present-day and Holocene populations, molecular data from Pleistocene fallow deer remain extremely limited, leaving a gap in our understanding of the species’ evolutionary history. Here, we retrieved 10 mitochondrial genomes from a ∼120-thousand-year-old last interglacial (Eemian) fallow deer population from the Neumark-Nord archaeological sites in central Germany. We find that modern European fallow deer throughout their western Eurasian distribution are closely related and form a single clade sister to the Eemian samples. Likewise, modern diversity is low compared to other cervids, whereas this single Eemian assemblage displays comparable levels to that of the entire modern range. Our results suggest that Neumark-Nord mitochondrial lineages were part of broader Late Pleistocene diversity, of which only one part appears to have persisted into the Holocene.

## Highlights

• 10 near-complete mitogenomes from 120,000-year-old fallow deer at Neumark-Nord
• Ancient local population is as diverse as the entire modern range
• Modern *Dama dama* forms a single clade sister to Eemian lineages
• Neumark-Nord morphological variation reflects within species local adaptation

## Graphical abstract

**Figure:**
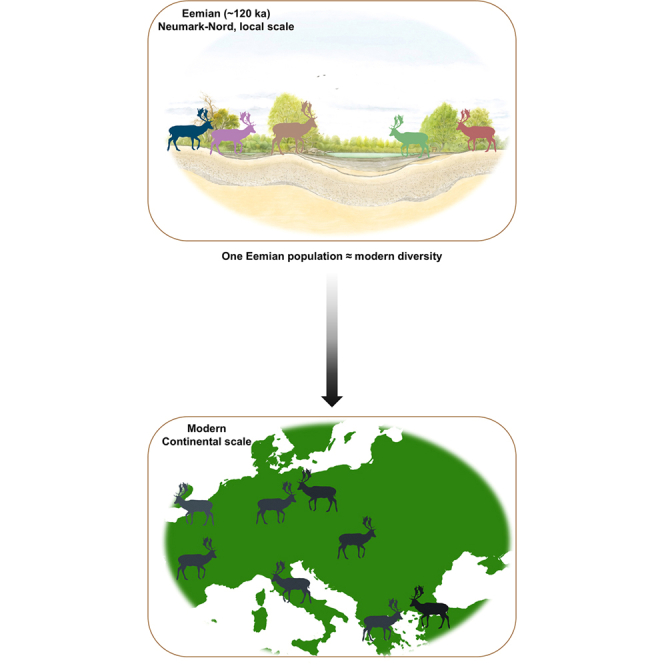

## Introduction

Within the Palearctic region, a key factor driving population divergence and ultimately species diversification is geography, along with climatic and environmental heterogeneity during the Quaternary.^1^^,^^2^ It is generally assumed that during glacial maxima, populations of warm-adapted species across Europe largely died out in northern latitudes, with only those already present in isolated southern refugia (e.g., Italy, Iberian Peninsula, Balkans) surviving.^3^^,^^4^^,^^5^ Moreover, some populations may have survived in cryptic northern refugia (i.e., sheltered valleys in limestone massifs that provided microclimates that favored the survival of thermophilic biotas).^6^ Both types of refugial populations likely expanded their ranges during interglacial periods as favorable climatic conditions returned.^1^^,^^7^^,^^8^ This scenario is supported by the fact that populations that inhabit regions that have been exposed to regular climatic oscillations tend to show low genetic variability,^9^ whereas higher genetic diversity is observed in populations from regions that have been historically stable.^6^ At the onset of the Mid-Pleistocene transition (from around 1.25 million years ago [Ma] to 0.7 Ma), Pleistocene glacial periodicity started to change from 40 thousand-year (ka) to roughly 100 ka cycles, fluctuating between extremely cold glacial stages and warm-temperate interglacials.^10^^,^^11^ During the middle Pleistocene, the Northern European Plain was therefore affected by permafrost conditions accompanying the glacial advances of the Elsterian (Marine Isotope stage [MIS] 10) and Saalian (MIS 6) glaciations.^12^^,^^13^^,^^14^ In contrast, during interglacial periods major parts of the Northern European Plain were covered with forests.^13^

One of the species affected by these climatic changes is the European fallow deer (*Dama dama* L. 1758), a medium-sized deer with well-established domestic and feral populations around the globe.^15^^,^^16^^,^^17^ *Dama* deer are considered to be the closest living relatives of the extinct giant deer or Irish elk (genus *Megaloceros*).^18^ Although previous taxonomic work has considered the fallow deer as a single species,^19^ more recent work has resulted in the recognition of two extant species; the Persian fallow deer (*D. mesopotamica*) and the European fallow deer (*D. dama*).^18^^,^^20^^,^^21^^,^^22^^,^^23^ Previous assessments of the European fallow deer’s modern and ancient origins and spread have been based on genetic, morphological or isotopic data including modern and ancient specimens that date from the Neolithic to the Roman period (ca. 9,000 to 1,600 years ago) as well as a single partial mitogenome from the British Eemian.^23^^,^^24^^,^^25^^,^^26^^,^^27^^,^^28^ As a result, Anatolia and the Balkans have been proposed as the two refugial areas where fallow deer survived the last glacial cold stage. Today, the province Antalya (Anatolia) represents the last region where a native herd survived after the original Balkan population went extinct around the early Middle Ages.^24^^,^^28^^,^^29^^,^^30^ However, based on genetic distances and geographic clustering, doubt has been cast on the proposition that modern populations have a single post-Roman Anatolian origin.^25^^,^^31^ In addition, zooarchaeological evidence suggests that the wide distribution pattern of the fallow deer observed today is a result of continuous human translocations since the Neolithic from southern source populations, as no populations of fallow deer are reported to have existed north of the Alps during the last glacial stage (MIS 4–2; 72–12 kya) or immediately afterward.^28^^,^^31^^,^^32^^,^^33^^,^^34^ Despite the efforts conducted thus far, a detailed examination of the evolutionary lineages and relatedness of ancient and modern fallow deer populations has been hampered by a lack of population genetic data from the Pleistocene, a time when the species was not yet severely affected by human translocation and management, even though there is evidence that individual fallow deer populations may have been severely affected by human hunting already during the Levantine Middle Paleolithic.^35^ A great number of sites with findings of fallow deer from the European Middle and Late Pleistocene interglacial periods have been described,^36^^,^^37^^,^^38^^,^^39^^,^^40^ and the existence of such sites suggests that the European fallow deer or at least close relatives were naturally widespread in Eurasia during the Eemian interglacial ∼125–115 ka.

Various locations in western and central Europe preserve evidence of large mammals with Mediterranean and African affinities that characterized the Eemian of central Europe (see ref.^24^^,^^40^ and SI Appendix Figure S1). Within Germany, Eemian sites have been reported in Weimar, Taubach, Belzig, Mannheim, Wiesbaden, and Neumark-Nord.^24^ The Neumark-Nord basins 1 and 2 are located in a region known as the “*Mitteldeutsches Trockengebiet*” (mid German dry area), which is characterized by being a relatively arid area with low precipitation regimes associated with the shadow cover created by the Harz mountains.^41^^,^^42^^,^^43^ The Neumark-Nord palaeobasins yielded undoubtedly one of the richest fossil assemblages that document the evolution of an endorheic basin (i.e., land locked drainage networks where water does not drain into large water bodies, and that experience water loss through percolation and evapotranspiration^44^) during a complete interglacial cycle. The Neumark-Nord basins were formed within a Saalian till and got filled with interglacial deposits in a fine-grained sedimentary matrix,^45^ which provides great opportunities for organic material preservation as well as a unique palaeoecological record of vegetation changes during the interglacial.^12^ Excavations of the high-resolution Neumark-Nord geological sequences have yielded several ungulate species such as rhinos, aurochs, cervids, and elephants, many recovered in association with stone artifacts and other archaeological materials which point to Neanderthal hunting.^41^^,^^42^^,^^43^^,^^45^^,^^46^^,^^47^ The unique preservation conditions have led to the recovery of the so far only multifold coverage nuclear genome from the Eemian outside the permafrost,^48^ from a straight-tusked elephant (*Palaeoloxodon*) specimen from Neumark Nord. We here focus on the European fallow deer (*Dama dama*) population inhabiting the Eemian lakeland of Neumark-Nord. The fallow deer from Neumark-Nord was first described by Pfeiffer^49^ as a subspecies of the extant *D. dama* (as *D. dama geiselana*) which shows clear morphological differences to other German and British fallow deer*.* Furthermore, a new taxonomic consideration^24^ proposed that this Neumark-Nord subspecies, as well as the one from the British Isles should even be upgraded to species level (*D. geiselana* and *D. clactoniana*, respectively) based on antler, skull, and teeth differences between them and modern fallow deer across Western Eurasia.

In this study, we employ ancient DNA analyses on the Eemian Neumark-Nord fallow deer population to address the following aspects: (1) to investigate the evolutionary history of fallow deer through the analysis of phylogenetic relationships between the ancient Neumark-Nord and modern Eurasian *Dama dama* populations, in order to evaluate the existence of distinct lineages within the species; (2) to analyze and compare genetic diversity estimates from modern and ancient European fallow deer populations, testing the hypothesis that ancient populations retained higher genetic diversity than their modern counterparts.

## Results

### Datasets

The investigated fossils were obtained from Neumark-Nord basin 1 and were assigned to *D. dama* (ssp. *geiselana*) based on their morphology. We collected and performed DNA extraction, library preparation, hybridization capture, and high-throughput sequencing on 44 European fallow deer fossil samples, 36 of which yielded *Dama* DNA sequences. From these, 10 samples were sufficiently preserved to yield a usable amount of mitochondrial DNA after hybridization capture (SI Appendix, Table S1). All our obtained ancient sequences show short fragment lengths and signals of cytosine deamination compatible with the high age of the specimens (SI Appendix, Figures S2 and S3). Completeness of our ancient mitochondrial genomes ranges between 75.58% (12,342 bp for sample DNN20) and 99.48% (16,246 bp for sample DNN29), with seven of them being >90% complete. These completeness values refer to the proportion of the mitochondrial reference genome recovered per sample. Likewise, we generated mitochondrial genome data from three additional modern fallow deer specimens from Germany (SI Appendix, Table S1).

### Genetic diversity

Genetic diversity measures in the European fallow deer reveal a consistent pattern of high haplotype diversity with low nucleotide diversity, observed both in modern and ancient populations (Table 1). When compared to four other cervid species (Figure 2), the European fallow deer stands out for its low mitochondrial diversity. The single ancient population from Neumark-Nord recovers a comparable level of diversity to that of all modern fallow deer originating from a much broader geographic distribution. In contrast, the giant deer (*Megaloceros* *giganteus*) and Sambar deer (*Rusa* *unicolor*) exhibit higher nucleotide diversity values than both of our fallow deer populations individually. Notably, the ancient red deer (*Cervus* *elaphus*) population from Cueva Liñares,^50^ dating to MIS3, also displays higher genetic diversity than the combined fallow deer populations. The higher diversity values observed in other widely managed species like the white-tailed deer (*Odocoileus* *virginianus*) and red deer emphasize the unique status of the European fallow deer. The recovered F_ST_ value of 0.65 between ancient and modern European fallow deer indicates substantial differentiation; however, given the temporal gap between the samples, this value reflects at least partially time dependent mutation accumulation, possibly in addition to demographic processes that affected the Eemian mitochondrial diversity (Table 1).

**Table 1:** Measurements of genetic diversity and demographic parameters for the European fallow deer samples analyzed in this study

Measurement	Ancient	Modern	*D. dama*
**Protein coding genes**			

Sample size	10	17	27
Total number of sites	6,859	10,588	5,629
Variable sites (S)	32	80	70
Haplotypes	9	12	20
Hd	0.978	0.949	0.969
Π	0.00126 ± 0.00025	0.0019 ± 0.00023	0.00263 ± 0.00022
F_ST_ ancient-modern	–	–	0.65341

**Full mitogenome**			

Sample size	10	17	27
Total number of sites	8,523	12,917	7,110
Variable sites (S)	32	92	70
Haplotypes	9	12	20
Hd	0.978	0.949	0.969
Π	0.001 ± 0.00021	0.00171 ± 0.00022	0.00209 ± 0.00018
F_ST_ ancient-modern	–	–	0.65409

Haplotype diversity (Hd), nucleotide diversity (π), and F_ST_ values that were obtained in DnaSP. Calculations were made considering both populations separately (ancient Neumark-Nord samples or modern) or joined together (depicted as *D. dama*). Total number of sites is calculated excluding sites with gaps and missing data.

### Phylogenetic relationships

The recovered ancient and modern fallow deer mitochondrial genomes were aligned with 38 additional cervid mitochondrial genomes (including 14 modern European fallow deer) retrieved from GenBank and subjected to maximum-likelihood (ML) and Bayesian inference (BI) phylogenetic analyses (see SI Appendix, Table S2). Phylogenetic trees were calculated on two different datasets, one using only protein coding genes plus the two ribosomal subunits (16 S and 12 S) obtained through mitochondrial annotation, and a second one which evaluates the protein coding genes, the two ribosomal subunits, and associated tRNAs (hereafter “full mitochondrial genome dataset”). The recovered median length for the protein coding genes dataset is 13,935 bp, whereas for the full mitochondrial genome dataset it is 15,436 bp. These values represent the total length of the alignments before filtering for missing or ambiguous sites. After filtering, the effective number of aligned sites retained for analyses ranges from 5629 to 12,917 bp, depending on the set of samples included (Table 1). As results did not differ between the protein coding genes and the full mitochondrial genome datasets, herein we only discuss the results obtained for the latter.

**Figure 1:**
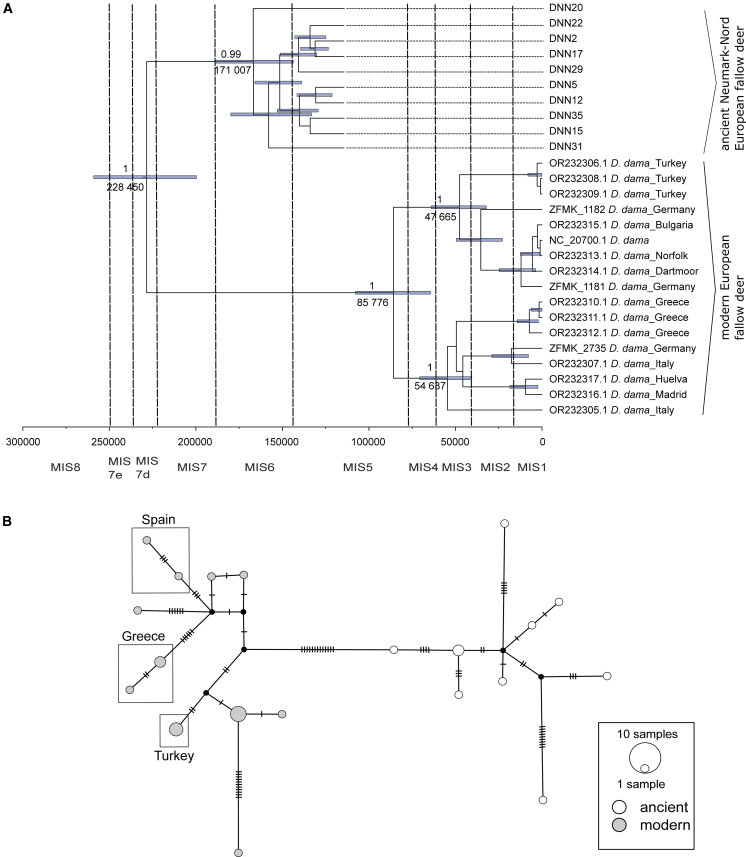
Evolutionary relationships between Eemian and modern fallow deer (A) Secondarily calibrated Bayesian phylogeny of 10 Eemian and 17 modern European fallow deer mitochondrial genomes. The root age was calibrated using the age estimate from the Bayesian mitochondrial species phylogeny. Branch values show posterior probabilities and median 95% HDP values from the best supported model, calibration set 3. Blue node bars represent the 95% credibility intervals of divergence times. (B) Median joining network of the mitochondrial genomes generated in POPART 1.7 considering positions that are present in all sequences while excluding ambiguities and missing data (alignment length: 7,110 bp), 70 variable sites. Color of haplotypes depict their origin: ancient Neumark-Nord samples (white), modern Eurasian samples (gray), and hypothetical inferred (black). Node sizes are proportional to the frequencies of the haplotypes. Bars on branches represent the number of mutational steps between the connected haplotypes.

Our phylogenetic analyses confirm the previously reported^18^^,^^20^^,^^21^^,^^22^^,^^23^^,^^25^^,^^26^^,^^27^^,^^30^ placement of *Dama* and *Megaloceros* as sister lineages with 100% bootstrap support (BS) and a Bayesian posterior probability (BPP) of 1. Likewise, we recover the distinction between the Persian fallow deer (*D. mesopotamica*) and the European fallow deer (*D. dama*) with 94% BS and 1 BPP, respectively. Although modern European fallow deer samples originate from the full current Eurasian distribution of the species (i.e., Germany, Spain, England, Italy, Greece, Bulgaria, and Turkey), they are genetically more closely related to each other (100% BS, 1 BPP) than to any of the ancient specimens and form a clade that is sister to the ancient samples from Neumark-Nord (Figures 1 and S1 Appendix Figures S4–S6). We reconstructed a median-joining haplotype network of the ancient and modern European fallow deer mitochondrial genomes (*n* = 27) with an alignment of 7,110 bp in length that showed 20 haplotypes with 70 segregating sites and a nucleotide diversity of 0.00209 (Table 1). Ancient and modern European fallow deer samples are separated by 13 mutational steps showing a reciprocally monophyletic pattern with no shared haplotypes. Geographic structure in modern fallow deer is weak, as illustrated by the grouping of samples from Germany, England, and Bulgaria in a single haplogroup that is separated from the Turkish specimens by only three mutations (Figure 1B). There is also close maternal relatedness between one German and one Italian individual (ZFMK 2735 and OR232307) as shown by a single mutational step between them. Consistent with this result, a median-joining network based on a 399 bp D-loop fragment spanning the Eemian, Roman, Medieval, and modern periods (see Baker et al.^26^) shows that the Neumark-Nord haplotypes are in a distinct and peripheral position relative to all post-Eemian sequences (SI Appendix, Figure S7). In contrast, Roman and Medieval haplotypes form central nodes surrounded by numerous derived variants, resulting in a pronounced star-like pattern consistent with population expansion and diversification during the Holocene. These combined results suggest that the modern fallow deer lineage represents only a subset of the mitochondrial diversity present in the Eemian, with the Neumark-Nord variation forming a distinct cluster relative to later European populations, consistent with the effects of long-term temporal separation. Furthermore, a manual inspection of the alignment identified six non-contiguous nucleotide positions at which 7–9 ancient individuals, with the respective positions not being covered in the remaining ancient individuals, share the same derived state, whereas all modern fallow deer retain the ancestral state observed in *D. mesopotamica*. This pattern is consistent with lineage-specific substitutions within the Neumark-Nord assemblage beyond the divergence expected from temporal separation alone. The high haplotype diversity among the ancient individuals implies either a comparatively large effective population size or the dispersal of multiple mitochondrial lineages into the Neumark-Nord region during the Eemian.

We applied a two-step time-calibrated Bayesian analysis to estimate the divergence times within the European fallow deer (*D. dama*) with different calibration points in order to account for inter- and intraspecific divergences (SI Appendix, Table S5). In the first step, the mitochondrial sequences of one ancient and two modern fallow deer were combined with those of closely related deer species to obtain primary dating results for the initial split within *D. dama* (hereafter termed the species phylogeny). Depending on the fossil calibration scheme (SI Appendix, Tables S5 and S6), the recovered 95% high density posterior means (HDP) for the divergence between European and Persian fallow deer ranged between 1.91 and 2.48 Ma, whereas the HDP for the most recent common ancestor (MRCA) of all European fallow deer mitochondrial genomes ranged between 130 and 228 ka, with the corresponding mean and median estimates being consistent for this split across phylogenies (SI Appendix, Figures S8–S11; Table S5). Our second calibration step was restricted to only include the European fallow deer individuals of this study (ancient and modern, hereafter the population phylogeny). We used twenty-seven mitochondrial genomes, excluding the control region (alignment length: 15,465 bp). Following the node age estimates for the fallow deer individuals analyzed in the species phylogeny, the root age of the population phylogeny was calibrated with three different inferred ages (calibration sets) using a normal prior as obtained from the MRCA from a Yule tree prior (mean 175 ka [95% HDP 138–216 ka]), the coalescent Bayesian skyline tree prior with no tip age (mean 175 ka [95% HDP 140–217 ka]), or a coalescent Bayesian skyline tree prior with tip dating (mean 228 ka [95% HDP 188–261 ka]). These three root ages were evaluated with two tree models to account for possible population fluctuations; thus, we used the coalescent constant size and coalescent exponential growth priors. Subsequent Bayes factors performed on the total six calibrations sets of the population phylogeny suggested that a constant size tree model with a root calibration with a mean of 228 ka (Bayesian skyline population model with tip dating, calibration set 3) best explained the data and performed slightly better than the constant size Yule tree prior (calibration set 1). In contrast, calibration set 2 received weaker Bayes factors support and was not preferred over either calibration sets 1 or 3. Calibration set 3 yielded a median coalescence time of 228 ka (95% HDP of 199–259 ka), whereas calibration set 1 yielded a median coalescence time of 202 ka (95% HDP of 174–229 ka) between ancient and modern fallow deer. Coalescence of Neumark-Nord samples had a range of 122–203 ka (calibration set 3) and 110 to 182 ka (calibration set 1), whereas modern samples coalescence is estimated to range from 64 to 107 ka and 62 to 100 ka, respectively (Figure 1A).

## Discussion

Our study provides population-level mitochondrial genomes of fallow deer (*D. dama* ssp. *geiselana*) with a completion of 75%–99% from an Eemian interglacial site in central Europe. Unlike permafrost regions, where cold conditions often favor DNA preservation, temperate environments pose significant challenges for the survival of ancient DNA over extended timescales. For instance, although Baker et al.^27^ retrieved a partial mitochondrial sequence from an Eemian fallow deer, the recovery of continuous, near-complete mitochondrial genomes from warm-temperate sites of this age remains challenging. The retrieval of partial mitochondrial genomes (75%–99% complete) from ten ∼120-thousand-year-old fossils provides a unique window into the last interglacial in central Europe and demonstrates the potential of suitable non-permafrost sites to preserve ancient DNA of this age. Using this molecular dataset, we were able to investigate the genetic identity of this population and provide insights into fallow deer evolution, its maternal genetic diversity and demographic history. Due to the different quality of samples and sequencing data (i.e., GenBank, ancient and modern samples obtained in this study), the amount of total information per sample varied; nevertheless, the overall patterns we observed do not differ between the different datasets we evaluated.

The inclusion of the Eemian samples from Neumark-Nord reveals that the Eemian population represents a deeper branch of diversity, of which only one lineage persists today. Although morphological differences have been reported in antler and tooth morphology that call for taxonomic separation,^24^ the Neumark-Nord specimens are still closely related to modern *D. dama* (Figures 1 and S1 Appendix). The morphological differences might thus just represent local adaptations or are part of the natural variation of the species.^51^ Thus, taxonomic separation of *D. d. geiselana* as a separate species is not warranted in our opinion. Furthermore, the recovered coalescence times on the population phylogenetic reconstructions show the associated difficulty in correlating phylogenetic dating with large-scale environmental fluctuations due to statistical uncertainty.^52^ Although the recovered node estimates within *D. dama* are broadly consistent (Figures 1 and S1 Appendix) despite the different calibrations used and indicate that modern and ancient European fallow deer diverged from one another sometime during MIS 6 or 7 (Table S5), they vary too much to conclusively decide whether the two major lineages diverged in a warm or cold phase during the Middle Pleistocene.

According to our best supported model, the recovered coalescence times on the population phylogeny show that the MRCA of the Neumark-Nord fallow deer predates the onset of the MIS 5 by ∼30 ka, with two additional lineage divergences overlapping this period (Figure 1A). This pattern is most parsimoniously explained if the observed genetic diversity in Neumark-Nord did not develop locally as a result of a small founder population but rather reflects the arrival of multiple pre-Eemian mitochondrial lineages, possibly reflecting a broader Late Pleistocene population that originated in distinct southern refugia during earlier glacial periods. Before the Eemian, *Dama* populations were restricted to the Mediterranean region, including Italy, Greece, and southern France (see in the study by Pfeiffer-Deml^24^). In this broader biogeographic context, during the Middle and Late Pleistocene, their expansion into Germany likely followed two primary migration routes that provide a plausible framework for understanding the origin of the observed diversity: an eastern route via the Danube valley, through the Balkans into the Rhume-Leine region and the Saale valley, with later dispersal to the Rhine valley during the Eemian; and a western Mediterranean route along the Rhone valley, spreading northwest to Britain, northern Germany, and the North Sea basin (see in the study by Pfeiffer-Deml^24^).

The reconstructed phylogenetic trees with the mitochondrial genomes of six genera within Cervini agree with previous studies showing a non-monophyletic genus *Rusa* and the placement of the giant deer (*M. giganteus*) as a sister species of the *Dama*-lineage.^18^^,^^53^ Furthermore, the relatively deep divergence between the Persian and European fallow deer is confirmed, with an estimated split of 2 Ma.^20^^,^^21^^,^^22^^,^^23^^,^^54^^,^^55^ Reliable estimates of divergence times are essential if we are to link divergence processes of *Dama* to geological events or climatic changes. Thus, the use of the fossil record as evidence plays an important role in estimating species and population divergence, given that uncertainty of a fossil’s age and placement can critically affect node age estimation.^56^^,^^57^^,^^58^ When fossil calibrations are placed on young nodes within a phylogeny, the estimated times tend to be underestimated, whereas with calibrations on ancient nodes, the ages of young nodes are likely to be overestimated.^59^ Thus, observable bias will have a direction toward the placement of the calibrations used. Here, we find that, despite using different sets of calibrations and tree priors, the divergence dates obtained show similar age ranges for the nodes evaluated in our species phylogeny (SI Appendix, Figures S7–S10, and Table S5). Paleontological evidence suggests that the earliest known record showing differentiation between the Persian and the European fallow deer dates to ca. 700 ka.^23^^,^^24^^,^^27^ However, all our estimated divergence times, which range from 1.74 to 2.79 Ma, suggest that the matrilineal lineage diversification of the fallow deer into Persian and European fallow deer substantially pre-dates this fossil evidence with limited variation between calibrations (Figures 1 and S1 Appendix Table S5). Since coalescent theory assumes that gene coalescence times predate species divergence times when gene flow is inexistent,^60^^,^^61^^,^^62^ our divergence dates describe the maximum age for maternal lineage divergence of fallow deer populations, at least in the absence of secondary gene flow.

The reconstructed haplotype networks show two reciprocally monophyletic groups with no evidence of mixing between them. We observe no shared haplotypes between the ancient and modern haplogroups, as expected given their temporal separation, and find no evidence of paraphyly in any of the reconstructed phylogenies. Similar to previous studies,^25^^,^^26^^,^^27^ we recover a clustering pattern in the modern fallow deer that shows weak geographic structure across Eurasia. The modern European fallow deer group is composed of two main clades. One clade is mainly represented by Turkish and northern European samples (including two of our reconstructed German fallow deer sequences), whereas the second mostly represents a Balkan-Iberian/Mediterranean group. Such clustering patterns have been regarded as recovering putative refugial populations.^23^^,^^27^^,^^30^^,^^31^ Since comparisons between the ancient and modern European fallow deer involve a separation of ca. 120 ka, during which some degree of mitochondrial divergence is expected to arise, the observed differences need to be interpreted relative to this temporal context. Our time-calibrated phylogenetic analyses and the presence of derived nucleotide states shared among multiple Neumark-Nord individuals suggest that the Eemian mitogenomes represent part of a Late Pleistocene genetic diversity broader than today’s. Moreover, our data provide no evidence that Neumark-Nord maternal lineages persisted after the Late Pleistocene, suggesting that they might have been lost during the last glacial (ca. 72–12 ka) (see in the study by Pfeiffer-Deml^24^). Modern fallow deer populations therefore appear to retain only a subset of this earlier mitochondrial DNA diversity.

**Figure 2:**
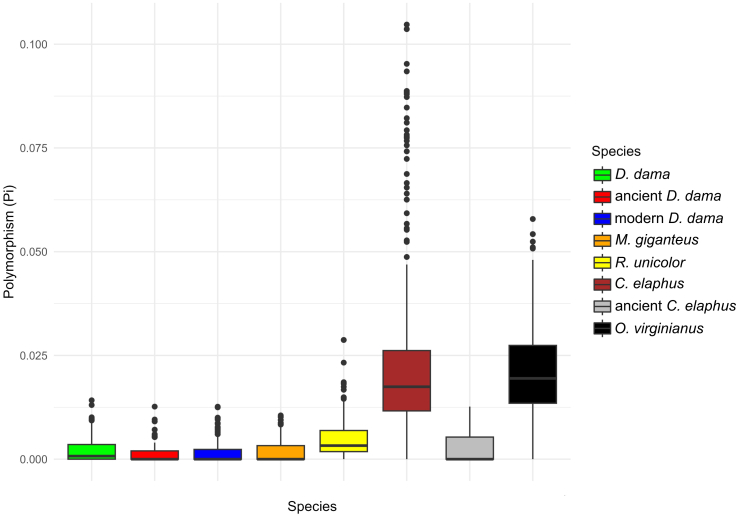
Boxplot for nucleotide diversity measurements across the mitochondrial genome for different cervids Comparisons were done both merging ancient (*n* = 10) and modern (*n* = 17) fallow deer samples (depicted as *D. dama*, *n* = 27) and considering each separately. Ten ancient *C. elaphus* individuals from Cueva Liñares,^50^ were used for comparison to a pre-LGM population. The number of samples is identical for the remaining species used (*n* = 11).

Our data show a striking feature of fallow deer population diversity across its Western Eurasian range, when considering modern and ancient populations. A single Eemian locality in central Europe recovers as much genetic diversity as that found in its modern counterpart with a Eurasian distribution originating from two hypothesized glacial refugia.^24^^,^^26^ Additionally, the recovered diversity from the modern fallow deer is remarkably low when compared with other extant species within Cervidae (Table 1; Figure 2), consistent with previous reports of low levels of variation within fallow deer populations (see^23^^,^^25^^,^^30^^,^^31^^,^^63^^,^^64^^,^^65^). This observed pattern provides insights into the demographic and evolutionary history of the species, suggesting the retention of many distinct, but closely related, mitochondrial lineages. Such patterns can be observed in species that are characterized by long-term stable populations that share occasional gene flow. Moreover, not only do the Sambar deer and giant deer exhibit higher diversity despite being sampled from a narrower geographic range; the fallow deer’s genetic variation remains remarkably constrained even when compared to a single ancient red deer population from Cueva Liñares.^50^ This comparison suggests that the observed low diversity in the European fallow deer reflects a species-wide pattern of reduced genetic variation that has been compounded by climatic and anthropogenic influences during the Late Pleistocene-Holocene.

Our observations agree with previous aDNA studies showing that Pleistocene populations often exhibit higher mitochondrial genetic diversity^66^^,^^67^^,^^68^^,^^69^^,^^70^^,^^71^^,^^72^ and suggest that a continent-wide sampling of Late Pleistocene fallow deer fossils would likely be more diverse than their modern counterpart. The low genetic diversity in modern populations may have resulted from population fragmentation into at least two, but possibly also more isolated groups during MIS cold stages, followed by the loss of multiple mitochondrial lineages, most likely during the glacial conditions of MIS 4–2. It is important to note that in the absence of nuclear data from fossil specimens, this scenario currently only applies to the maternally inherited mtDNA and hence, to the female lineage. Female fallow deer are often philopatric^24^^,^^73^ thereby limiting female-mediated genetic exchange across fragmented populations. At the population level, philopatry can become a detrimental strategy when fragmentation-driven reduction of populations threatens population viability. For instance, the behavior of mammal species such as the Bottlenose dolphin (*Tusiops truncatus*),^74^^,^^75^ the Pink river dolphin (*Inia boliviensis*),^76^ the Greater mouse-eared bat (*Myotis myotis*),^77^ plateau pikas (*Ochotona curzoniae*),^78^ and others,^79^^,^^80^^,^^81^ collectively illustrate how philopatry has led to vulnerable populations with hindered adaptability in the face of environmental change. Finally, the previously described inability of fallow deer populations to track suitable habitats^27^^,^^82^ furthermore has the potential for compounding the effects of philopatry and fragmentation during cold Pleistocene periods. Similar patterns of historical fragmentation and subsequent loss of mitochondrial genetic diversity due to the inability of tracking suitable habitats have been observed in other species, such as the Arctic fox (*Alopex lagopus*) or the Saiga antelope (*Saiga tatarica*).^66^^,^^83^

The study of ancient DNA is a valuable tool to provide insights into the evolutionary relationships and population dynamics between extinct and extant lineages.^84^^,^^85^ It has been pointed out before that caution is warranted in the interpretation of single genetic loci, as the stochastic nature of the genealogical process can affect inference from such loci^86^; nevertheless, given the associated difficulties of recovering DNA material from ancient samples^87^ mitochondrial DNA remains to be the marker of choice for many ancient DNA studies, especially outside the permafrost. In the case of the European fallow deer, we identified two distinct lineages and found that the species likely possesses historically low mitochondrial diversity compared to other cervids. Although the Neumark-Nord assemblage spans approximately 2,000 years, representing ca. 300–400 fallow deer generations, this interval is expected to produce negligible mitochondrial divergence (∼0.004% or ca. one substitution across the analyzed alignment). It is therefore treated here as an effectively contemporaneous population snapshot. Remarkably, this single Eemian locality in central Europe (Neumark-Nord) recovers as much genetic diversity as that found in all modern populations, despite the latter having a much wider geographic distribution. This finding suggests a loss of diversity in modern fallow deer during or after the last glacial stage, which led to minimal geographic structuring and to the strongly reduced genetic diversity observed in modern populations.

### Limitations of the study

An important merit of our study is that it provides a rare population-level genetic view of a last interglacial (Eemian) ecosystem, demonstrating that high-quality ancient DNA can be recovered from a ca. 120 ka deposit outside the permafrost. This represents a significant advance for reconstructing deep-time population dynamics in temperate regions. However, as is typical for ancient DNA studies, our ability to recover genetic information is limited by sample preservation. Our conclusions are based on a relatively small number of individuals from a single locality, which limits the resolution of population-level patterns and restricts our ability to assess broader demographic trends across the species’ range. Furthermore, few archaeological sites of comparable age, context, and preservation conditions are likely to yield material of similar quality, limiting opportunities for broader spatial comparison. Although the recovered mitogenomes provide meaningful insight into Late Pleistocene fallow deer diversity, expanded datasets from additional well-preserved last interglacial contexts will be essential to determine whether the patterns observed at Neumark-Nord are regional or more widespread across Eemian fallow deer populations.

## Resource availability

### Lead contact

Requests for further information and resources should be directed to and will be fulfilled by the lead contact, Michael Hofreiter (michael.hofreiter@uni-potsdam.de).

### Materials availability

This study did not generate new unique reagents. All specimens analyzed belong to the Landesamt für Denkmalpflege und Archäologie Sachsen-Anhalt-Landesmuseum für Vorgeschichte located in Halle (Saale) and are currently curated by the Archaeological Research Center and Museum for Human Behavioral Evolution (Archäologisches Forschungzentrum und Museum für menschliche Verhaltensevolution), MONREPOS, Germany; under the catalog numbers listed in the supplemental information, Table S1. Access is subject to the repository’s policies.

### Data and code availability

• Data: The data supporting the findings of this study are available within the paper. Raw sequencing data have been deposited at the NCBI SRA Portal and are publicly available as of the date of publication. The accession link is listed in the key resources table. The mitochondrial sequences for the Neumark-Nord fallow deer samples have been deposited in GenBank under the following accession numbers: PX395816–PX395828.
• Code: This paper does not report original code.
• Other items: Any additional information required to reanalyze the data reported in this paper is available from the lead contact upon request.

## Acknowledgments

We are grateful to Harald Meller and the Landesamt für Denkmalpflege und Archäologie Sachsen-Anhalt-Landesmuseum für Vorgeschichte, the owner and responsible authority of the Neumark-Nord material for constant support and encouragement, granting research permissions and access to the find material. We thank Michaela Preick and Silke Abelt for their support in laboratory work. We thank all the “AlterEco” group members for valuable discussions regarding the archaeological material. We acknowledge everyone involved in collecting the samples studied. We also thank the Leibniz Institute for the Analysis of Biodiversity Change (LIB) and M. Sc. Laura von der Mark, data manager of the Biobank, for providing us the three German fallow deer samples used in this study. This study was made possible through financial support from the Leibniz Zentrum für Archäologie, the 10.13039/501100001664Leibniz Association (Collaborative Excellence Funding “AlterEco: Understanding the “Anthropocene”: human alteration of ecosystems in our deep history” (K283/2019) to S.G.-W. and L.K.), Deutsche Forschungsgemeinschaft (DFG Grant GA 683/7-1 to S.G.-W.), Fellowship “Zielgerade” by 10.13039/501100004812Gutenberg Forschungskolleg of Johannes Gutenberg-University Mainz (to S.G.-W.), 10.13039/501100003246Netherlands Organization for Scientific Research (Spinoza Grant 28-548 to W.R.), and 10.13039/501100001717Leiden University’s Liveable Planet Program (to W.R.).

## Author contributions

Conceptualization, A.R.-M., P.A., L.K., and M.H.; methodology and data curation, A.R.-M., P.A., M.H., L.K., S.G.-W., and W.R.; investigation and formal analysis, A.R.-M., P.A., and M.H.; writing—original draft, A.-R.M., P.A., and M.H.; writing – review and editing, A.R.-M., P.A., L.K., S.G.-W., W.R., F.S., and M.H. All authors gave final approval for publication.

## Declaration of interests

The authors declare no conflict of interest.

## STAR★Methods

### Key resources table

**Table:** 

REAGENT or RESOURCE	SOURCE	IDENTIFIER
**Chemicals, peptides, and recombinant proteins**		

Guanidine hydrochloride	Roth	Cat#0037.1
QIAGEN MinElute kit	QIAGEN	Cat#28004
Sodium hypochlorite, reagent grade	Sigma Aldrich	Cat#425044
Analytical reagent grade Potassium hydroxide	Fisher Scientific	CAS No: 1310-58-3
Ethylenediaminetetra-acetic acid	VWR Chemicals	CAS No: 60-00-4
HPLC grade Sodium acetate trihydrate	Fisher Scientific	CAS No: 6131-90-4
Sodium azide	VWR Chemicals	CAS No: 26628-22-8
HPLC grade Methanol	Fisher Scientific	CAS No: 67-56-1

**Critical commercial assays**		

D1000 Screen Tape (Tapestation2200)	Agilent	Cat #5067-5582
dsDNA HS Assay Kit (Qubit 2.0)	Thermofisher	Cat#Q32851

**Deposited data**		

Raw sequencing data	BioProject ID: PRJNA1454232	http://www.ncbi.nlm.nih.gov/bioproject/1454232
Mitochondrial genome sequences	This paper	GenBank: PX395816–28

**Oligonucleotides**		

CL9 extension primer: GTGACTGGA GTTCAGACGTGTGCTCTTCCGATCT	Gansauge et al.^88^	Sigma Aldrich
Double-stranded adaptor	Gansauge et al.^88^	Sigma Aldrich
Strand 1 (CL53): CGACGCTCTTC-ddC (ddC = dideoxycytidine)	–	–
Strand 2 (CL73): [Phosphate]GGAAGAG CGTCGTGTAGGGAAAGAG∗T∗G∗T∗A (∗ = phosphothioate linkage)	–	–
CL78: AGATCGGAAG[C3Spacer] _10_ [TEG-biotin] (TEG = triethylene glycol spacer)	Gansauge et al.^88^	Sigma Aldrich
P5 indexing primer: AATGATACGGCGAC CACCGAGATCTACACnnnnnnnnACACT CTTTCCCTACACGACGCTCTT	Gansauge et al.^88^	Sigma Aldrich
P7 indexing primer: CAAGCAGAAGACG GCATACGAGATnnnnnnnnGTGACT GGAGTTCAGACGTGT	Gansauge et al.^88^	Sigma Aldrich
IS7 amplification primer: ACACTCTTTCCCTACACGAC	Gansauge et al.^88^	Sigma Aldrich
IS8 amplification primer: GTGACTGGAGTTCAGACGTGT	Gansauge et al.^88^	Sigma Aldrich
CL72 R1 sequencing primer: ACACTCT TTCCCTACACGACGCTCTTCC	Gansauge et al.^88^	Sigma Aldrich

**Software and algorithms**		

Cutadapt v3.4	Martin^89^	https://cutadapt.readthedocs.io/en/stable/
BWA v0.7.17-r1188	Li and Durbin.^90^; Li and Durbin.^91^	http://bio-bwa.sourceforge.net/
Samtools v1.15	Li et al.^92^	https://sourceforge.net/projects/samtools/files/samtools/
MapDamage v2	Jónsson et al.^93^	https://ginolhac.github.io/mapDamage/
MAFFT v7.480	Katoh and Standley^94^	https://mafft.cbrc.jp/alignment/server/index.html
MitoFinder v1.4.1	Allio et al.^95^	https://github.com/RemiAllio/MitoFinder
AliView 1.28	Larsson^96^	https://ormbunkar.se/aliview/
PopART 1.7	Leigh et al.^97^	https://popart.maths.otago.ac.nz/download/
DnaSP v6	Rozas et al.^98^	https://www.ub.edu/dnasp/
RaxML v8.2.12	Stamatakis^99^	https://cme.h-its.org/exelixis/web/software/raxml/
BEAST v1.10.4	Drummond et al.^100^	https://beast.community/
Partitionfinder v2.11	Lanfear et al.^101^	https://github.com/brettc/partitionfinder
Tracer v1.7.2	Rambaut et al.^102^	http://tree.bio.ed.ac.uk/software/tracer/
Treeannotator v1.10.4	Drummond et al.^100^	https://beast.community/treeannotator
FigTree v1.4.2	N/A	http://tree.bio.ed.ac.uk/software/figtree/

**Other**		

Proteinase K	Promega	Cat#V3021
FastAp	Thermo Fisher	Cat#EF0651
Dynabeads MyOne C1	Thermo Fisher	Cat#65001
T4 DNA Polymerase	Thermo Fisher	Cat#EP0061
Buffer Tango (10×)	Thermo Fisher	Cat#BY5
T4 DNA ligase	Thermo Fisher	Cat#EL0011
Accuprime Pfx	Thermo Fisher	Cat#12344024
PEG-4000	Thermo Fisher	Cat#EP0061
Klenow fragment of DNA polymerase I	Thermo Fisher	Cat#EP0051
SYBR green PCR MasterMix	Thermo Fisher	Cat#4309155

### Experimental model and study participant details

#### Archaeological samples

In 1985, during the German Democratic Republic (GDR) era, first bone fragments and articulated skeletons of several ungulate species were uncovered in the Eemian basins of Neumark-Nord 1 and 2^41^^,^^42^^,^^43^^,^^45^ in the former lignite open cast mine Mücheln. Among the most remarkable finds were remains of straight-tusked elephants, aurochs and also cave lions and hyenas. The Neumark-Nord site is characterized by a unique preservation of organic material that documents in great detail a long history of environmental changes during the Eemian interglacial. Additional specimens were uncovered during active mining and subsequent reclamation works until 2009, and through several multidisciplinary studies, the deposits were dated to around 132 ± 12 kyrs.^41^^,^^103^^,^^104^ More than 100 articulated skeletons and partial skeletons of fallow deer or close relatives were recovered in excellent condition from these lake sediments. Their morphology and variation in bone proportions and dimensions were thoroughly examined and differentiated from those of *Cervus* and other close relatives.^49^^,^^105^

We collected bone samples from 44 fallow deer individuals found in the Neumark-Nord basins (SI Appendix, Figure S1; Table S1). Sampled material ranged from 70 to 200 mg of bone pieces (mainly M3 teeth). A section of approximately 2 cm^2^ from the fossil M3 teeth was cut with an electric rotary drill (Dremel, Racine, WI, USA). When more bone material from the same individual was available, samples were also collected from the inner ear and/or mandible bones (SI Appendix, Table S1), as dense material has been shown to conserve more endogenous DNA.^87^^,^^106^ All specimens analyzed belong to the *Landesamt für Denkmalpflege und Archäologie Sachsen-Anhalt-Landesmuseum für Vorgeschichte* located in Halle (Saale), and are currently located at the Archaeological Research Centre and Museum for Human Behavioural Evolution (*Archäologisches Forschungzentrum und Museum für menschliche Verhaltensevolution*), MONREPOS, Germany.

#### Modern samples

We obtained three modern fallow deer samples from two localities in Germany, namely the municipality of Hausen and the Hochwildpark Rheinland in Nordrhein-Westfalen. Samples were provided to us as DNA extracts by the Leibniz Institute for the Analysis of Biodiversity Change (LIB).

### Method details

#### Ancient DNA extraction and sequencing

All ancient DNA extractions were performed in a designated ancient DNA laboratory at the University of Potsdam including negative controls for both extraction and library preparation. Each batch of DNA extractions included two negative controls processed alongside bone samples using identical reagents and conditions. DNA concentration in the blanks was assessed with a Qubit 2.0 Fluorometer (Thermo Fisher Scientific, Waltham, US-MA) using the dsDNA Assay kit. When no DNA was detected, 10 μl of each blank were combined and used as a library blank. After sequencing, reads from negative controls were processed in parallel with our sample data; all controls produced negligible (< 250) numbers of reads, confirming the absence of detectable contamination. Additionally, blank libraries were screened for potential contamination by mapping reads against common laboratory contaminants and the human genome, with no significant hits detected. Bone samples were first crushed using a mortar and pestle to obtain smaller fragments. Afterwards, bone fragments were powdered in 50 ml RETSCH MM 400 Mixer Mill grinding jars (#1.4112; Verder Scientific). We used ∼50 mg of bone powder for overnight digestion at 37°C in 1 ml extraction buffer (0.25 mg/ml Proteinase K in 0.45 M EDTA) with constant rotation. After digestion, DNA was purified following the Dabney et al.^107^ protocol with minor modifications. In brief, undigested material was pelleted using centrifugation and the supernatant was transferred into 13 ml binding buffer (5 M guanidine hydrochloride, 40% isopropanol, 0.005% Tween-20, and 90 mM sodium acetate). The mix was passed through QIAGEN MinElute columns fitted with a reservoir. Two consecutive washing steps of the silica membrane were done using 650 μl Prerinsing and Elution (PE) buffer (QIAGEN) followed by a dry spin to remove any PE buffer remnants. The purified DNA was eluted in 25 μl TET buffer (10mM Tris-HCL, 1mM EDTA, 0.05% Tween-20). From these extracts, a maximum of 13 ng DNA was used as input for library construction. Protocols from Gansauge et al.^88^^,^^108^ were followed for library preparation. In short, we removed uracil residues, which tend to accumulate in ancient DNA as a result of cytosine deamination,^109^ using USER® Enzyme (New England Biolabs) in a 21 μl reaction. One unit of FastAP (Thermo Fisher Scientific, Waltham, US-MA) was used to remove residual phosphate groups and the DNA was denatured at 95°C for 2 minutes. We performed the ligation of the first adapter in 80 μl reactions with the following reagent concentrations: 50% (vol/vol) PEG-8000, ATP 100 nM, Splinter Oligo (10/20 μM), and T4 DNA ligase (30 U/μl). Ligation products were then immobilized on streptavidin covered magnetic beads (Dynabeads) to allow the removal of reagent mixtures in subsequent steps. The extension primer CL130 was then annealed to the complementary oligo sequence and the strand complementary to the template single-stranded molecules filled-in using Klenow fragment (10 U/μl) in 50 μl reactions. The second double-stranded adaptor (CL53/CL73) was then ligated to the blunt-end molecules in a 100 μl reaction containing 1x T4 DNA ligase buffer, 5% (vol/vol) PEG-4000, 0.025% (vol/vol) Tween 20, 2 mM double-stranded adaptor, and 0.1 U/μl T4 DNA ligase. The library strand complementary to the original single-stranded template molecule was then released by heat denaturation and eluted in 25 μl TET buffer. Next, libraries were amplified using AccuPrime Pfx DNA polymerase (Thermo Fisher Scientific, Waltham, MA) and indexed (using unique indices within both P5 and P7 adapters) in 80 μl reactions. Prior to amplification, optimal PCR cycle numbers were established by quantitative PCR (PikoReal Real-Time PCR, Thermo Fisher Scientific) in 10 μl with primers IS7 and IS8.^88^ Based on the amplification curves, libraries were amplified using between 13 and 19 cycles to achieve sufficient yield while minimizing over-amplification and PCR duplicates. The amplified and indexed libraries were then quantified on a TapeStation 2200 (Agilent Technologies, Santa Clara, US CA) using a D1000 screen tape and on a Qubit 2.0 Fluorometer (Thermo Fisher Scientific, Waltham, US-MA) using the dsDNA Assay kit. Indexed samples were pooled in equimolar ratios for test sequencing on an Illumina NextSeq500 system (Illumina, San Diego, US-CA) at the University of Potsdam.

After test sequencing, the libraries that yielded sufficient endogenous DNA concentrations were used for hybridization capture. In total, 10 samples were enriched for mitochondrial DNA by performing two rounds of in-solution hybridization capture using MyBaits capture probes (ArborBioscience) and following the MyBaits-manual-v3 with modifications in temperature and salt concentrations.^110^ Published mtDNA genome sequences of *Dama dama* (NC_020700.1) and *Cervus elaphus* (NC_007704.2) were used for the design of the probes. A qPCR (PikoReal Real-Time PCR, Thermo Fisher Scientific) was used to determine the cycles necessary for post-capture amplification. The amplified post-capture libraries were then quantified using a TapeStation 2200 (Agilent) and a D1000 screen tape, followed by Qubit 2.0 Fluorometer measurement using the dsDNA Assay kit. Finally, all samples were pooled in equimolar ratios for sequencing on an Illumina NextSeq500 system (Illumina, San Diego, US-CA) generating ca. 10M 75 bp single-end reads per sample.

#### Modern sample amplification and sequencing

The DNA extracts were first evaluated for DNA concentration and fragment size lengths on a TapeStation 2200 (Agilent Technologies, Santa Clara, US CA) using a genomic tape. After evaluation, extracts were sheared to a target size of 500 bp using a Covaris S220 System (Covaris, Woburn, US-MA) and thereafter converted into double-stranded, double indexed Illumina sequencing libraries.^111^^,^^112^ Briefly, we conducted blunt-end repair of the extracted DNA in 35 μl reactions containing 1x Buffer Tango, 100 μM each dNTP, 1 mM ATP, 0.5 U/μl T4 Polynucleotide Kinase, 0.1 U/μl T4 Polymerase and 25 μl template DNA. Following incubation and inactivation at 25°C and 72°C, respectively, for 20 minutes each, double-stranded adaptors were ligated in a 60 μl reaction that contained 1x T4 DNA Ligase Buffer, 5% (w/v) PEG-4000, 0.125 U/μl T4 DNA Ligase and 0.5 μM double-stranded adaptor mix. After incubating at 22°C for 30 minutes, the products were purified using the QIAGEN MinElute kit. Appropriate indexing PCR cycles were determined with a quantitative PCR (PikoReal Real-Time PCR, Thermo Fisher Scientific). The resulting libraries were purified using the QIAGEN MinElute kit, and quantified on a TapeStation 2200 (Agilent Technologies, Santa Clara, US CA) with D1000 screen tape and reagents, and a Qubit 2.0 Fluorometer (Thermo Fisher Scientific, Waltham, US-MA) using the dsDNA Assay kit. Sequencing was performed on the same Illumina platform at the University of Potsdam with the indexed samples pooled in equimolar ratios for 1M 75 bp reads per sample.

#### Mitochondrial sequence data processing

Single-end sequencing data in raw fastq format were merged and quality inspected in Fastqc.^113^ Afterwards, Cutadapt v3.4^89^ was used to trim adaptor sequences and low-quality bases (< Q30); sequences with read length <30 bp were also discarded. Trimmed reads were mapped to the mitochondrial reference genome of *Dama dama* (NC_020700.1) with the program bwa v0.7.17- r1188^90^^,^^91^ using the aln algorithm and reducing the allowed difference to n=0.01. We then used samtools v1.15^92^ to remove reads with a mapping quality below 30. Duplicate reads (reads with the same start and end coordinates) were identified and removed with samtools “rmdup”. To corroborate the ancient origin of our DNA samples, cytosine deamination patterns and read length distribution were calculated using MapDamage v.2 (SI Appendix, Figures S2 and S3).^93^ A consensus sequence was called using the bcftools v1.11^114^ mpileup command and only regions with a minimum read depth of 3x were kept. The most complete consensus sequence recovered from mapping to the fallow deer reference (DNN29) covers a length of 16,246 bp (99.48% complete) with an overall coverage of 13.4x. The sample with the least recovered length used in this study (DNN20), has a sequence length of 12,342bp (75.58%) with a coverage of 3.93x. The three modern German fallow deer samples were quality inspected and mapped to the species reference as described before to obtain consensus sequences with a minimum read depth of 3x; from these three samples we recover ∼94% of the mitogenome (SI Appendix, Table S1).

#### Phylogenetic and genetic diversity analyses

We downloaded a set of complete mitochondrial genomes from GenBank. Sequences corresponded to different cervid species from the Cervinae subfamily and one representative from the Capreolinae that was used as outgroup (SI Appendix, Table S2). The resulting GenBank data set was combined with our generated fallow deer sequences and an initial alignment was produced with MAFFT v7.480.^94^ We annotated our generated data set with Mitofinder v1.4.1^95^ using the Megahit v1.0^115^ and MITFi algorithms^116^ and produced separate alignments for each mitochondrial coding gene. Alignments were visually curated, refined and trimmed to the first base of a codon for protein coding sequences in AliView 1.28.^96^ The resulting gene alignments were concatenated with AMAS.^117^ Partitioning of the data set for each codon position was analyzed with PartitionFinder v2.11^101^ with a greedy search algorithm, considering all the available models for MrBayes and selecting the most appropriate model based on the BIC criterion. A recent analysis of the European fallow deer genetic structure has made available a partial mitochondrial sequence (5,354 bp) from an Eemian sample of the English site Joint Mitnor Cave, Devon (Ipswichian dated based on faunal content).^27^^,^^36^ This ancient Eemian sequence was obtained through the supplementary materials provided by the authors and was then annotated with the program Mitofinder v1.4.1.^95^ Given that the published sequence is highly fragmented, we attempted to find segments that could be aligned with our data set. We identified fragments of the ND1 (391 bp), COX1 (501 bp), and 16S rRNA (386 bp) genes. After conducting exploratory phylogenetic analyses including this partial sequence (data not shown), we discarded it for further analyses given its fragmented nature, which ultimately resulted in too limited overlap with our dataset.

In total, phylogenetic trees were calculated using maximum likelihood (ML) and Bayesian inference (BI) approaches with two different data sets: one using only protein coding genes (with a total of 13 genes) together with the sequences of the 12S and 16S ribosomal RNA (rRNA) units obtained through annotation (hereafter “protein coding genes” alignment), and a second data set where the protein coding genes, the two rRNA units, and associated tRNAs were evaluated (hereafter “full mitogenome” alignment). Both the protein coding genes alignment and the full mitogenome alignment were done with the control (D-loop) region removed, due to repetitive sequences, and alignment ambiguities.^118^^,^^119^

We used the program RaxML v8.2.12^99^ to calculate a ML phylogenetic tree using the GTRCAT substitution model for all the partitions. We estimated nodal support via 1,000 bootstrap replicates. The resultant partition schemes and model substitution parameters were also used for conducting the phylogenetic reconstruction with the BI approach, as implemented in MrBayes v3.2.6.^120^ We ran two independent searches using four Markov-Chains Monte Carlo for 10^7^ generations and sampling every 1,000th step. Convergence across runs was evaluated using two methods: a) by examination of the standard deviation of split frequencies (with acceptance values <0.01); and b) by verification of parameter estimates in Tracer v1.7.2,^102^ based on acceptable effective sample sizes (ESS values > 200). After checking for convergence, the first 25% of the generated trees were discarded as burn-in and the remaining 75% were kept for calculation of posterior probabilities.

For network construction, we used the fallow deer sequences available in both data sets. This resulted in a data set consisting of 27 sequences. We used PopArt 1.7^97^ to visualize the relationships among all haplotypes represented in each data set by constructing networks using the median-joining algorithm,^121^^,^^122^ assigning equal weights to all variable sites and an epsilon parameter with default values (ε=0). Additionally, we constructed a network using the aligned regions of the D-loop from our three most complete samples (DNN2, DNN22, DNN29) together with a set of sequences from Baker et al.^26^ This alignment was 399 bp in length and included 62 sequences. Genetic diversity measures such as haplotype diversity (Hd), nucleotide diversity (π) and FST values were calculated in DnaSP^98^ for the protein coding and full mitogenome data sets. We assessed the significance levels of the tests via the generation of 1,000 coalescent simulations under a neutral model.

#### Divergence time estimation

To estimate the divergence time between the Neumark-Nord fallow deer and the remaining analyzed cervids, a Bayesian MCMC-based approach was used as implemented in BEAST 1.10.4.^100^ First, we tested whether each of our data sets fits either to a strict clock model or to a relaxed clock model and performed selection tests through the stepping-stone method (SS)^123^ as implemented in MrBayes v3.2.6.^102^ Given the partitioning models obtained in PartitionFinder (SI Appendix, Table S3), the mean marginal likelihood of a strict clock performed better than a relaxed molecular clock in both of our data sets (SI Appendix, Table S4). Afterwards, we adopted a two-step strategy for the calibrated analyses. We generated fossil-calibrated phylogenies using different sets of combinations of three fossil dates and tree priors (SI Appendix, Tables S5 and S6). In short, fossil calibrations are based on the oldest *Rusa* fossil (3.4–2.6 Ma)^53^^,^^124^; the *D. mesopotamica* and *D. dama* divergence (700 ka ± 50 ka)^24^^,^^27^; and finally using the earliest possible occurrence of *Megaloceros giganteus* of ca. 450 ka (± 100 ka).^125^ Given the congruence between our two data sets, we selected 13 sequences representing the different cervid species, including two modern fallow deer and our most complete ancient fallow deer sample (DNN29) from the full mitogenome data set. For representation of the modern fallow deer, we kept for this analysis the reference sequence (NC_020700) and an individual from Turkey (OR232306). Hereafter, this alignment is called the species alignment to distinguish it from the alignment with only fallow deer sequences, which is called the population alignment. A set of phylogenies were estimated through a Yule speciation and coalescent Bayesian Skyline tree model with a UPGMA starting tree under the strict clock and using the substitution models previously selected by PartitionFinder for the full mitogenome data set with chains run for 10^7^ generations. We set a mean clock rate value of 1.65 x 10^-8^ subs/site/year with a standard deviation of 0.01 based on previous Artiodactyla studies.^126^^,^^127^ Lognormal age distributions were chosen for our three fossil priors^128^; likewise for all age priors the minimum age of the oldest known fossil was used as the offset parameter. When using the coalescent Bayesian skyline tree prior, we also set the age of the Neumark-Nord sample to a conservative 120 ka. This age reflects the stratigraphic context where the fallow deer remains were found, which were mainly recovered from layer 7 (dated to ca. 120 ± 6 ka).^12^^,^^41^^,^^104^ This layer corresponds to the Upper Gyttja of Mesocratic Phase 2 (*Carpinus-Picea* phase) within the Eemian. Chain convergence and parameter sampling were examined by eye using Tracer v.1.7.2.^102^ The first 25% of samples were discarded from each run, and trees were summarized and maximum clade credibility trees (MCCT) with median node heights were annotated using TreeAnnotator v.1.10.4.^129^ Node ages are represented as mean heights and 95% credibility interval values (CI) with a posterior probability limit of 0.6. Node ages with no CI reported had a lower probability. FigTree v1.4.2 (http://tree.bio.ed.ac.uk/software/figtree/) was used for visualization of the resulting trees.

To investigate the influence that different sets of priors have on divergence time estimates, the root age estimates for the fallow deer individuals analyzed in the species phylogeny was alternatively set and evaluated as follows: a) The root age was calibrated to the MRCA of the fallow deer as obtained from the Yule tree prior; setting the root age for the fallow deer MRCA as obtained from the coalescent Bayesian skyline tree prior with no tip age added; or finally, setting the fallow deer MRCA as obtained from the coalescent Bayesian skyline tree prior with tip dating. b) The effect of data set composition was investigated by using two population alignments (protein coding genes, full mitogenome) each with the calibrations previously mentioned. Each model calibration was evaluated using two tree prior models, i. e. coalescent constant size and coalescent exponential growth. Parameters for the models such as starting tree, type of molecular clock or number of MCMC chains run were the same as described above. Adjustment of the data sets to each of the calibrations used was evaluated using Bayes factors (BF), calculated from the marginal likelihoods from the stepping-stone (SS) method in BEAST 1.10.4.^100^^,^^129^ Each marginal likelihood was estimated through 100 path steps with a Beta distribution (03, 1.0). We considered 3 Log ml differences as strong evidence against the null hypothesis.^130^^,^^131^ The MCCT was evaluated, generated, and visualized as described above.

### Quantification and statistical analysis

All statistical analyses and phylogenetic inferences were performed using the software packages described in the method details section. Statistical details for each analysis, including sample sizes, parameters, and models used, are provided throughout the Methods, Results, figure legends, and supplementary tables.

Maximum likelihood phylogenetic analyses were conducted in RAxML v8.2.12 using 1,000 bootstrap replicates. Bayesian phylogenetic analyses were performed in MrBayes v3.2.6 with two independent runs of four Markov chains Monte Carlo for 10^7^ generations, sampling every 1,000 generations. Convergence was assessed using split frequency values and ESS values in Tracer v1.7.2. Genetic diversity statistics were calculated in DnaSP with significance assessed through 1,000 coalescent simulations. Divergence time analyses were performed in BEAST v1.10.4 under the models and parameters described above. For sequencing analyses, coverage statistics and sequence completeness are reported in the corresponding sections and supplementary tables.
