# Supplementary material for: Eemian palaeogenetics demonstrates loss of diversity in modern fallow deer (Dama dama)
Source: iScience. 2026 Jun 3;29(6):116204. doi: 10.1016/j.isci.2026.116204 (PMC13255044; doi:10.1016/j.isci.2026.116204)
Supplement: Document S1. Figures S1–S12 and Tables S1–S4 [file mmc1.pdf]

iScience, Volume *29*

Supplemental information

Eemian palaeogenetics demonstrates loss of diversity in modern fallow deer (*Dama dama*)

Alberto Rocha-Méndez, Patrick Arnold, Lutz Kindler, Sabine Gaudzinski-Windheuser, Wil Roebroeks, Fulco Scherjon, and Michael Hofreiter

Additional Results

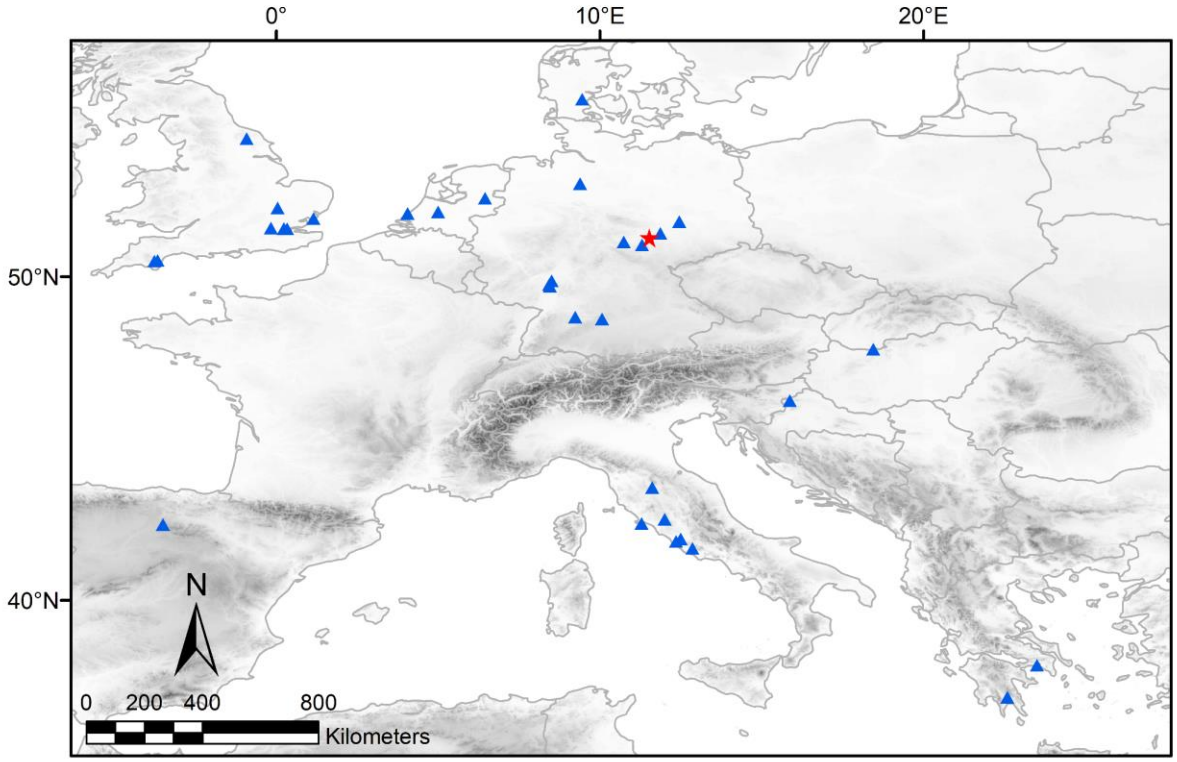

Figure S1. Overview of Pleistocene *Dama* fossil distribution, Related to STAR Methods. Map showing the localities where Pleistocene *Dama* and fossils from closely related deer have been found throughout western Europe (blue triangles). Records have been obtained from the NOW community (https://nowdatabase.luomus.fi/) and Pangaea (https://www.pangaea.de/) data bases. The Neumark-Nord site, from where the samples used in this study derive, is shown as a red star.

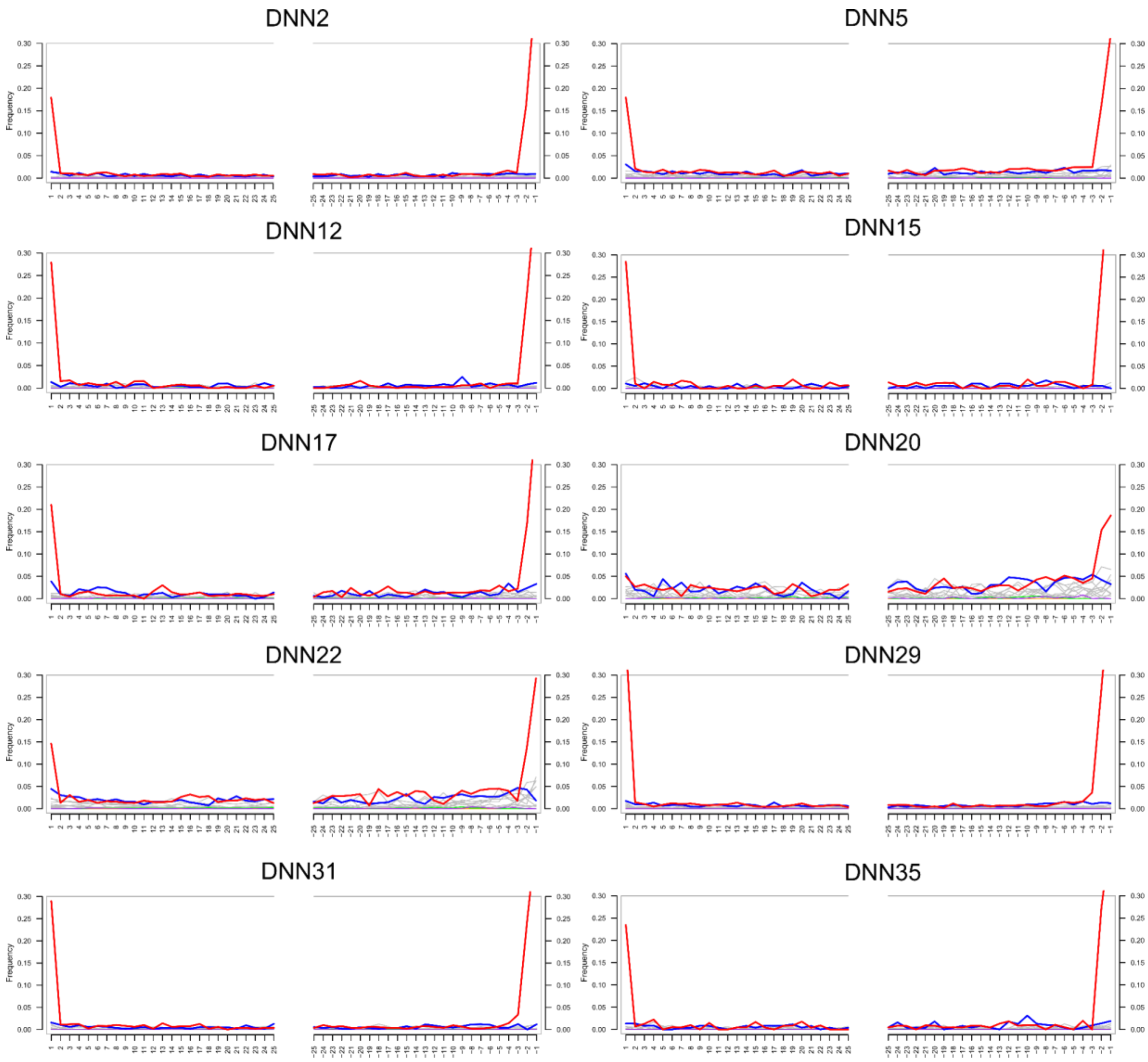

Figure S2. DNA authentication, Related to STAR Methods. Frequency of C to T misincorporations (red), and G to A (blue) from the 5’ (left) and 3’ (right) end for reads mapping to the *D. dama* reference.

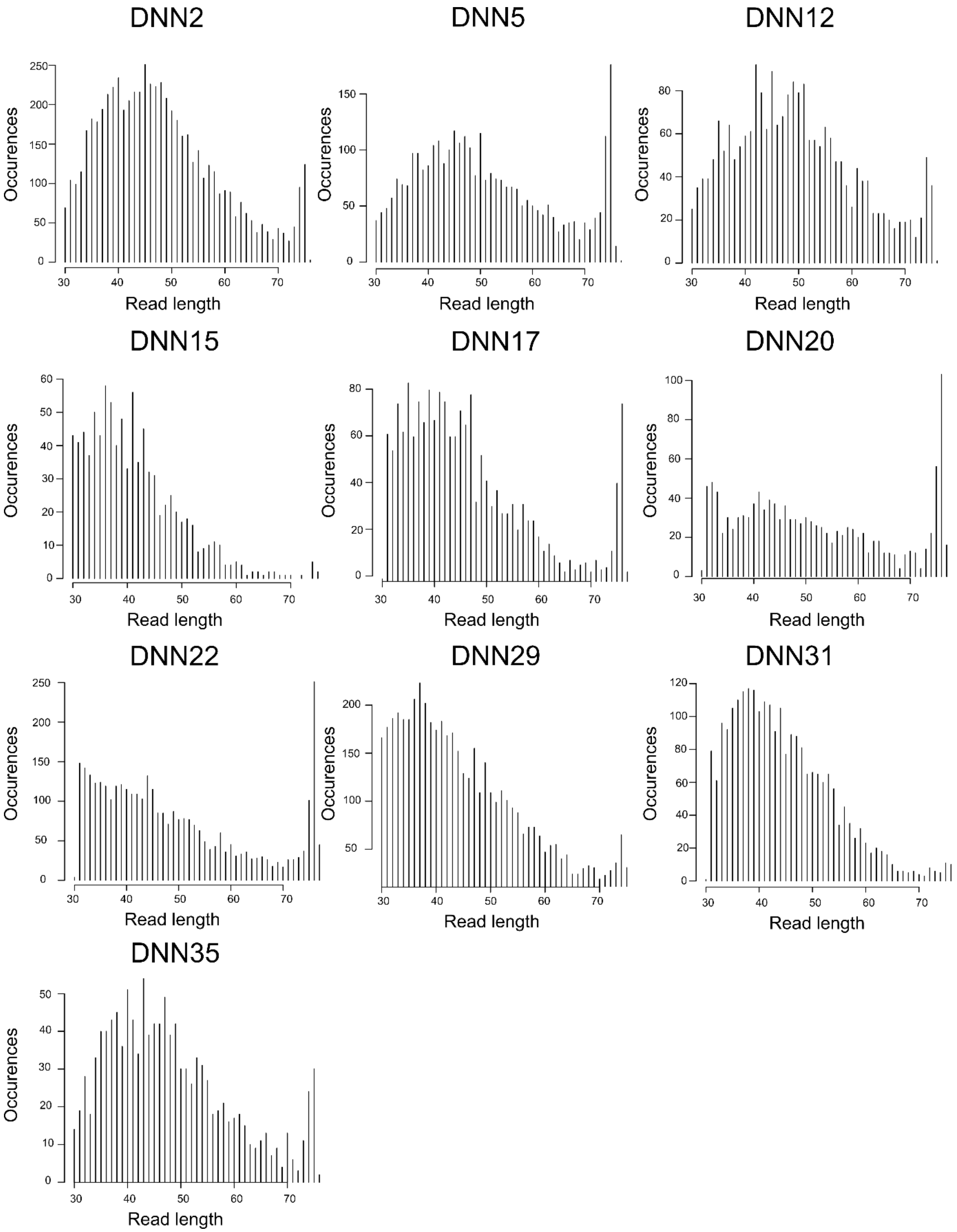

Figure S3. DNA authentication, Related to STAR Methods. Single-end read length distribution for reads mapping to the *D. dama* reference.

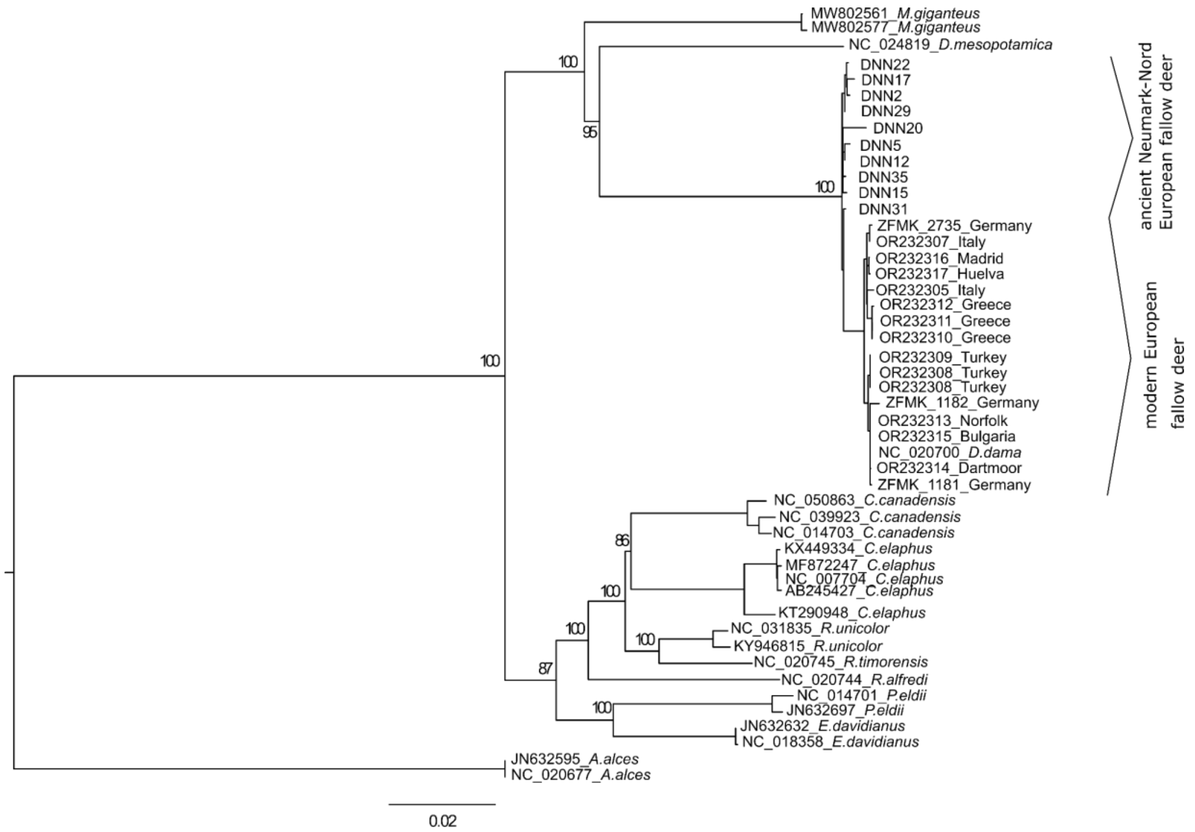

Figure S4. Protein coding gene phylogeny, Related to STAR Methods. Maximum likelihood phylogeny obtained for the protein coding genes. Node support is given as bootstrap support values.

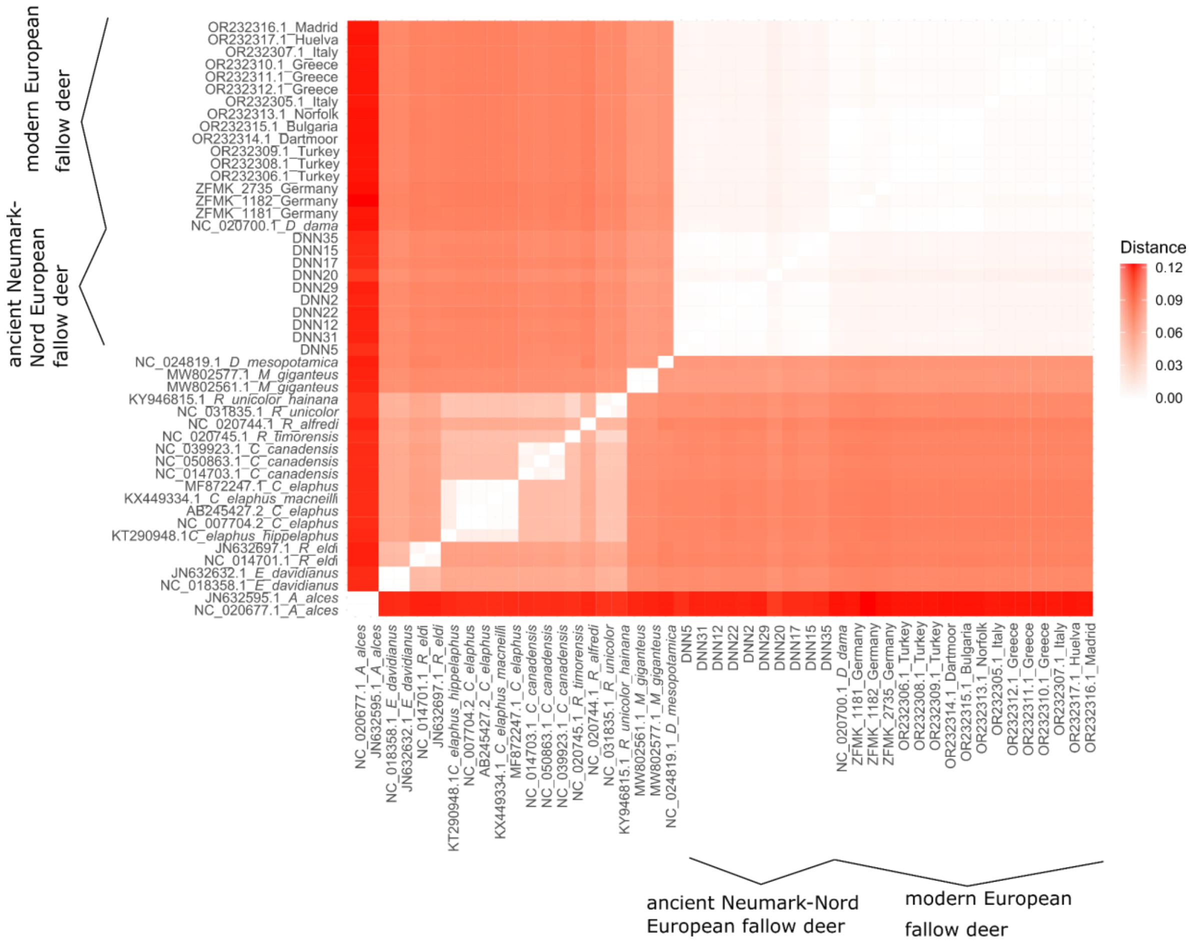

Figure S5. Distance matrix heatmap based on pairwise distance matrix from the full mitogenome data set, Related to STAR Methods. The matrix was calculated with a Neighbor-Joining algorithm and a Kimura-two-parameter evolutionary model. Distance values range from 0 to 0.12 and correspond to a gradient of color steps that range from white (lowest distance value) to red (highest distance value).

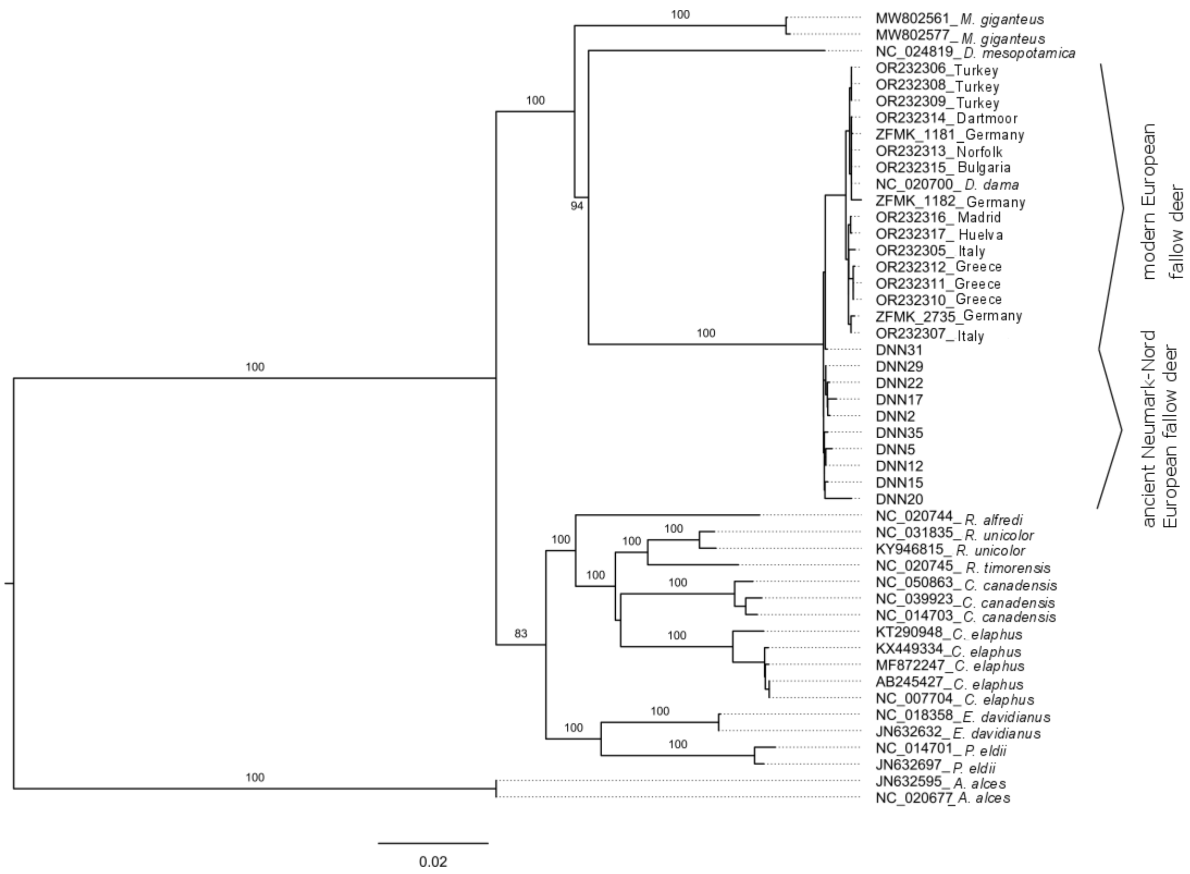

Figure S6. Full mitochondrial genome maximum likelihood phylogeny, Related to STAR Methods. Node support is given as bootstrap support values. Bootstrap values under 80 are not shown.

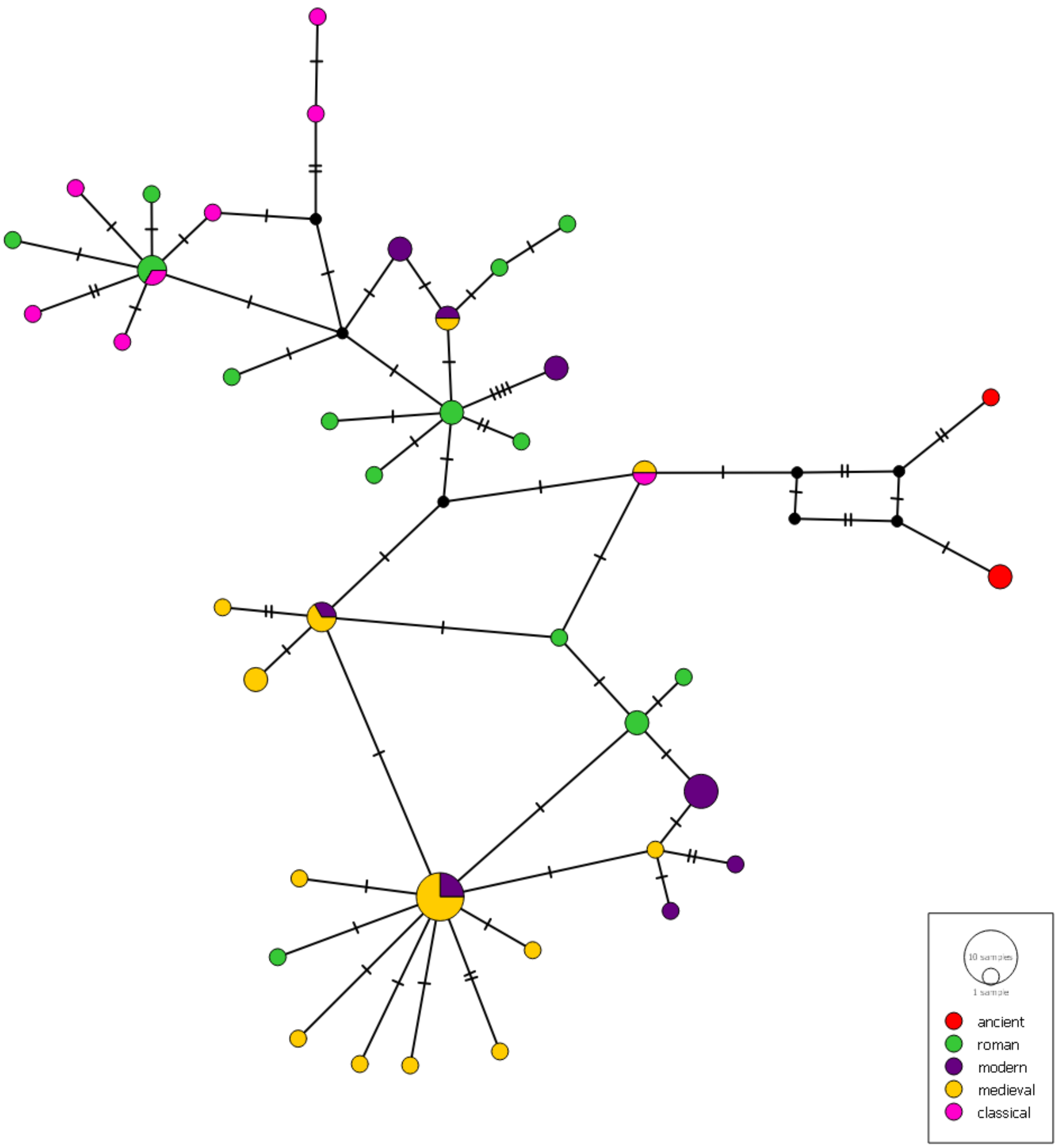

Figure S7. Median-joining haplotype network of ancient, historical and modern fallow deer mitochondrial partial D-loop sequences, Related to Figure 1B and STAR Methods. The network was constructed using 399 bp alignment of the mitochondrial D-loop region including three ancient fallow deer specimens from Neumark-Nord (red) and published sequences from Baker et al. (see ref. 26) encompassing distinct time periods herein depicted as Classical (5th century before our current era - 1st century; pink), Roman (2nd-6th century; green), Medieval (7th-16th century; yellow), and modern (17th century to present). Each circle represents a unique haplotype, with circle size proportional to haplotype frequency. Bars on branches represent the number of mutational steps between the connected haplotypes; whereas black circles represent hypothetical haplotypes that were not sampled. The Neumark-Nord haplotypes form a distinct cluster separated by multiple substitutions from other groups, while Roman and Medieval clusters display a star-like pattern consistent with demographic expansion.

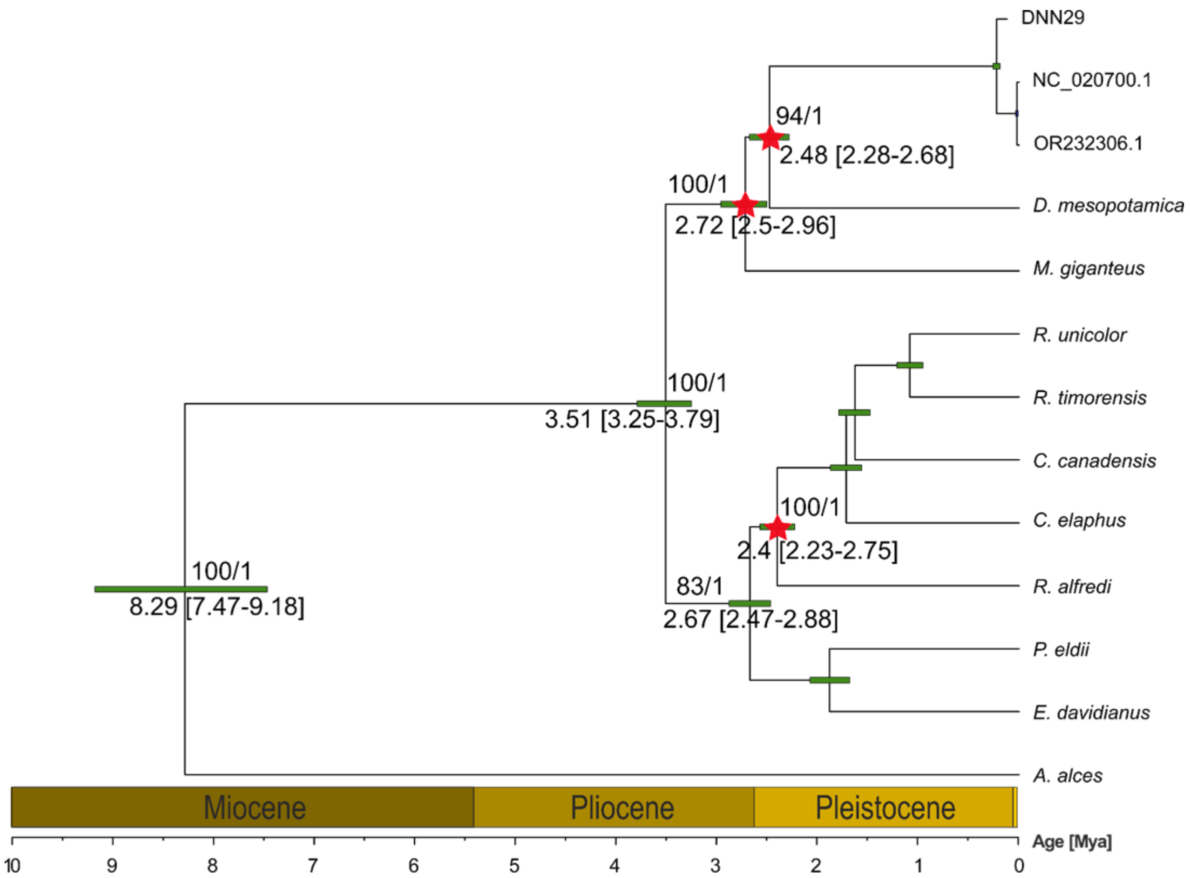

Figure S8. Dated tree obtained for the species phylogeny with calibration set 3, Related to STAR Methods. Values above branches depict bootstrap support followed by the Bayesian posterior probabilities. Values below branches show median coalescence ages. Numbers in brackets show 95% HDP age estimates. Green bars show the 95% highest posterior density estimates of divergence times for nodes with a posterior probability above 0.6. Red stars in the phylogeny show the fossil calibration points used.

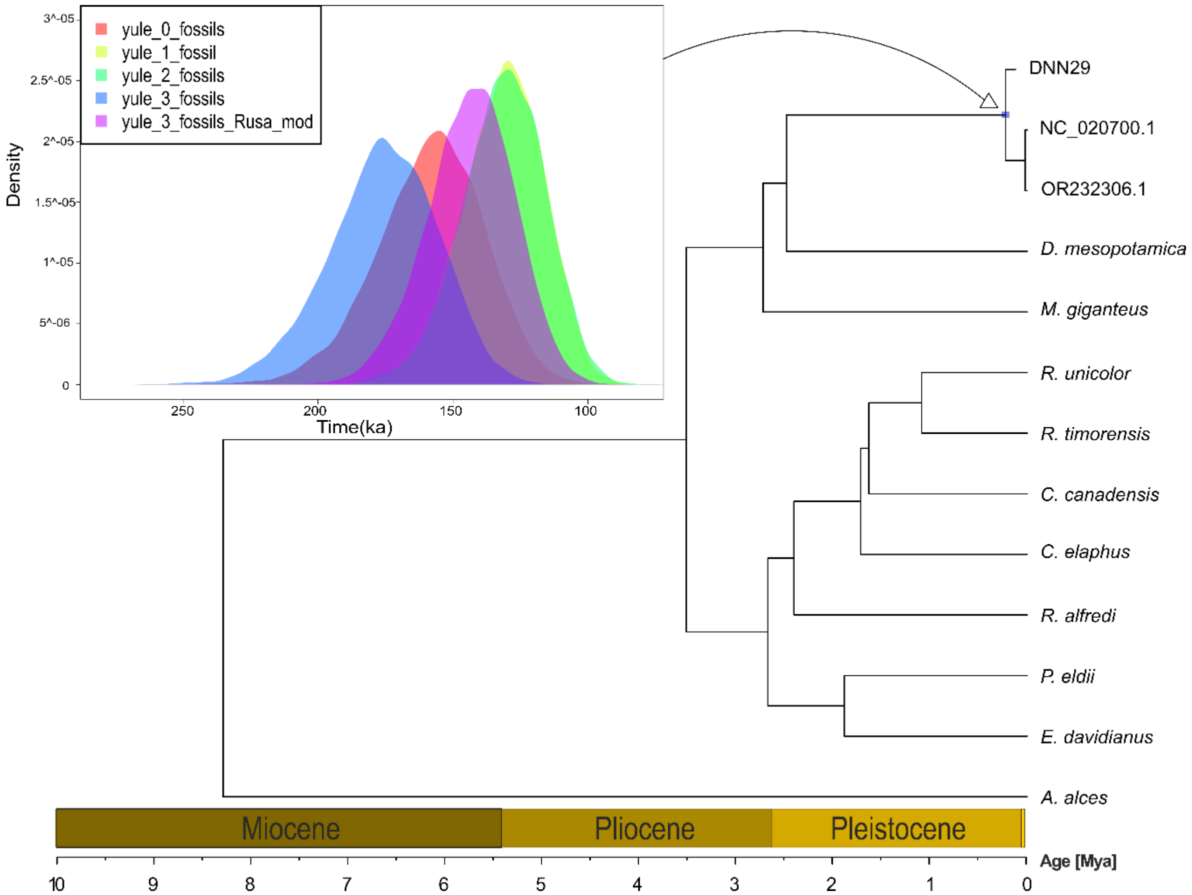

Figure S9. Calibrated mitochondrial species phylogeny of 13 deer sequences, Related to STAR Methods and Table S5. The inset shows the density distribution of divergence times for the different calibrations using none to three fossil calibrations and a Yule tree prior. Node support of Bayesian posterior probability for all recovered nodes is 1. When legend includes “Rusa_mod”, this indicates that the oldest fossil calibration was moved to the crown node for evaluating the influence on the divergence estimate.

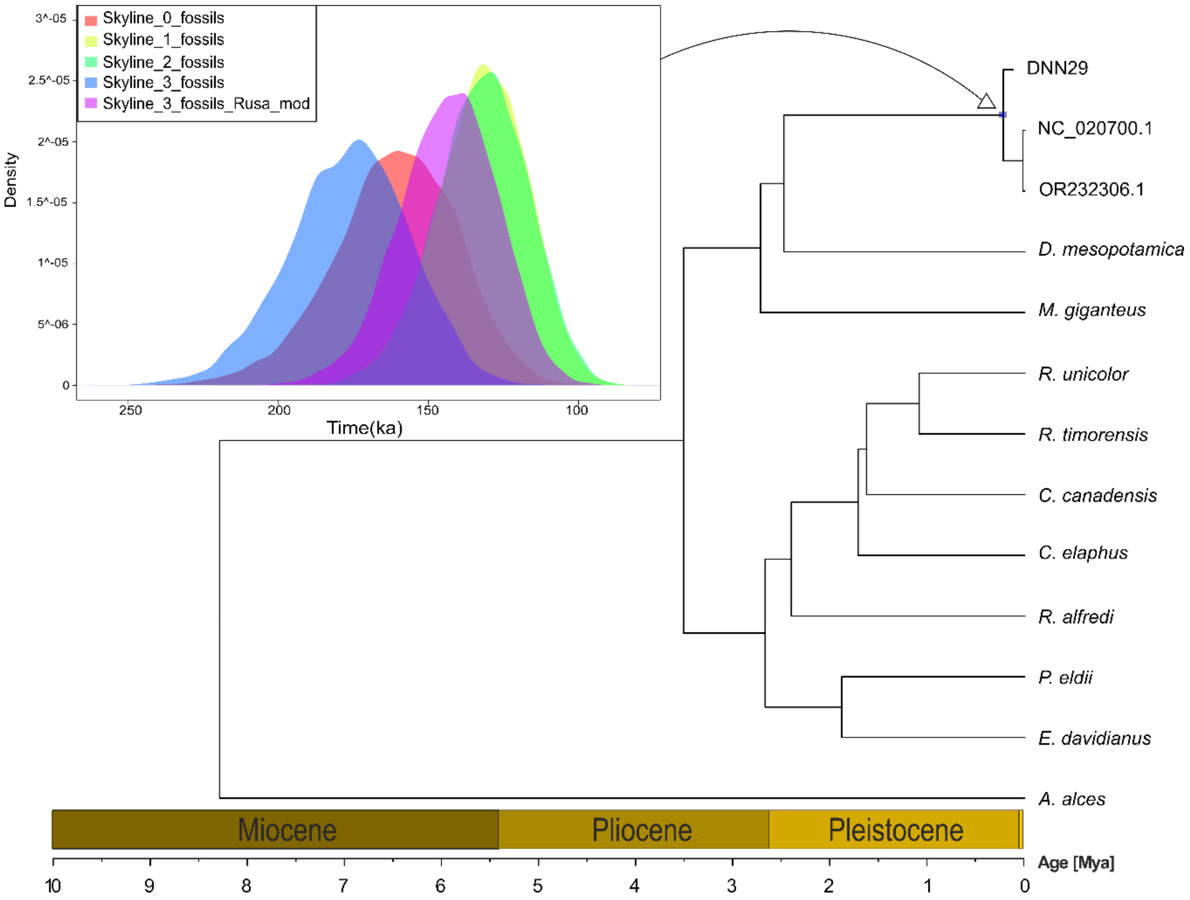

Figure S10. Calibrated mitochondrial species phylogeny of 13 deer sequences, Related to STAR Methods and Table S5. The inset shows the density distribution of divergence times for the different calibrations using none to three fossil calibrations and a Bayesian Skyline tree prior. Node support of Bayesian posterior probability for all recovered nodes is 1. When legend includes “Rusa_mod”, this indicates the oldest fossil calibration was moved to the crown node for evaluating the influence on the divergence estimate.

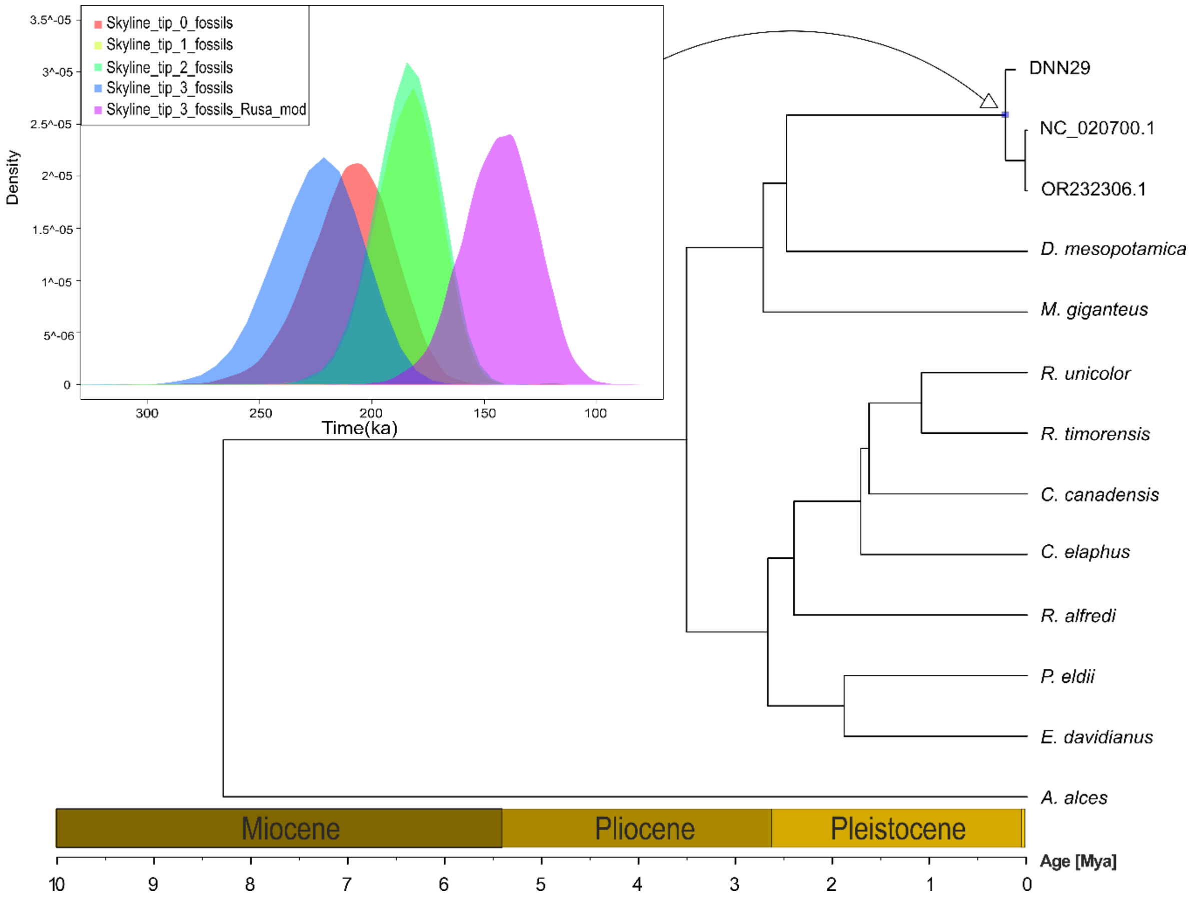

Figure S11. Calibrated mitochondrial species phylogeny of 13 deer sequences, Related to STAR Methods and Table S5. The inset shows the density distribution of divergence times for the different calibrations using none to three fossil calibrations, tip ages for the Neumark-Nord samples, and a Bayesian Skyline tree prior. Node support of Bayesian posterior probability for all recovered nodes is 1. When legend includes “Rusa_mod”, this indicates the oldest fossil calibration was moved to the crown node for evaluating the influence on the divergence estimate.

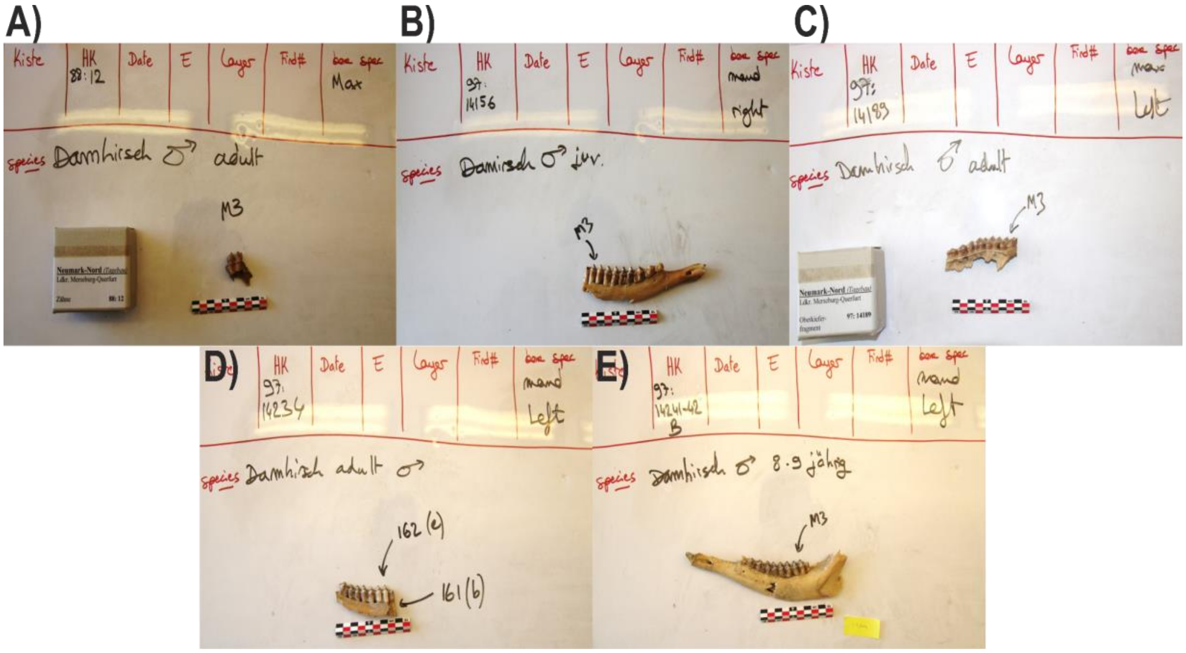

Figure S12. Sampled skeletal elements from five of our sequenced specimens in this study. Related to STAR Methods. See table S1 for sample details. A) Sampled M3 tooth corresponding to DNN29. B) Sampled M3 tooth with right mandible corresponding to DNN12. C) Sampled M3 tooth with left mandible corresponding to DNN35. D) Sampled M3 tooth with left mandible showing two places from which samples were taken that correspond to DNN22. E) Sampled M3 tooth and left mandible corresponding to DNN15

Table S1. Dama samples with mapping statistics, Related to STAR Methods. Details for archaeological samples with associated mapping statistics using the *D. dama* mitochondrial genome (NC_020700) as reference. The last three samples correspond to the modern German fallow deer samples provided by the Leibniz Institute for the Analysis of Biodiversity Change (LIB).

Sample code	Skeletal element	Museum identifier	Mappable reads	Mapped reads	Unique mapped reads	Mapped bp	Duplication	Covered percentage of the mitogenome	Mean coverage (X-fold)
**DNN2**	Petrous bone	HK97: 14154	3,922,650	57,186	6,095	292,233	0.11	98.70	17.9x
**DNN5**	Petrous bone	HK97: 14172	2,552,869	139,198	3,221	164,309	0.02	95.68	10.1x
**DNN12**	Tooth fragment M3	HK97: 14156	11,120,800	7,176	2,215	109,415	0.31	96.7	6.7x
**DNN15**	Tooth fragment M3	HK97: 14241-42(B)	17,918,621	17,800	912	37,441	0.05	82.79	2.29x
**DNN17**	Petrous bone	HK97: 14192	14,411,337	19,105	1,864	84,648	0.1	90.52	5.18x
**DNN20**	Tooth fragment	HK87: 300, 960-961	12,652,956	679,891	1,268	64,213	0.0018	75.58	3.93x
**DNN22**	Tooth fragment M3 / Petrous bone	HK97: 14234	60,292,962	1,306,956	3,567	171,055	0.002	98.739	10.5x
**DNN29**	Tooth fragment M3	HK88: 12, 76-77	17,088,652	14022	4,870	218,874	0.35	99.48	13.4x
**DNN31**	Tooth fragment	HK97: 14175 (92:1000)	13,607,670	17,446	2,465	106,955	0.14	95.82	6.55x
**DNN35**	Tooth fragment M3	HK97: 14189	13,935,407	3201	1,204	57,742	0.38	87.8	3.54x
**ZFMK_1181**	n.a.	ZFMK-DNA-155631181	346,266	779	658	45,258	0.84	93.99	3.2x

**ZFMK_1182**	n.a.	ZFMK-DNA-155631182	785,371	2091	1431	48,015	0.68	94.51	3.72x
**ZFMK_2735**	n.a.	ZFMK-DNA-FC19442735	615,623	1002	673	45,423	0.67	93.74	3.54x

Table S2. Genbank sequences, Related to STAR Methods. Complete mitochondrial genome sequences downloaded from Genbank that were used in this study and their associated source.

Complete mitogenomes		
**Species**	**GenBank accession number**	**Source**
Dama dama	NC_020700.1	Hassanin, A., *et al*., 2012. https://doi.org/10.1016/j.crvi.2011.11.002
	JN632629.1	Hassanin, A., *et al*., 2012. https://doi.org/10.1016/j.crvi.2011.11.002
	OR232309.1	Baker, K.H., *et al.,* 2024. https://doi.org/10.1038/s41598-023-48112-6
	OR232308.1	Baker, K.H., *et al.,* 2024. https://doi.org/10.1038/s41598-023-48112-6
	OR232306.1	Baker, K.H., *et al.,* 2024. https://doi.org/10.1038/s41598-023-48112-6
	OR232314.1	Baker, K.H., *et al.,* 2024. https://doi.org/10.1038/s41598-023-48112-6
	OR232315.1	Baker, K.H., *et al.,* 2024. https://doi.org/10.1038/s41598-023-48112-6
	OR232313.1	Baker, K.H., *et al.,* 2024. https://doi.org/10.1038/s41598-023-48112-6
	OR232312.1	Baker, K.H., *et al.,* 2024. https://doi.org/10.1038/s41598-023-48112-6
	OR232311.1	Baker, K.H., *et al.,* 2024. https://doi.org/10.1038/s41598-023-48112-6
	OR232310.1	Baker, K.H., *et al.,* 2024. https://doi.org/10.1038/s41598-023-48112-6
	OR232307.1	Baker, K.H., *et al.,* 2024. https://doi.org/10.1038/s41598-023-48112-6
	OR232317.1	Baker, K.H., *et al.,* 2024. https://doi.org/10.1038/s41598-023-48112-6
	OR232316.1	Baker, K.H., *et al.,* 2024. https://doi.org/10.1038/s41598-023-48112-6
Dama mesopotamica	NC_024819.1	Hassanin, A., *et al*., 2012. https://doi.org/10.1016/j.crvi.2011.11.002
Megalocerus giganteus	MW802561.1	Rey-Iglesia, A., *et al.,* 2021. https://doi.org/10.1098/rspb.2020.1864

	MW802577.1	Rey-Iglesia, A., *et al.,* 2021. https://doi.org/10.1098/rspb.2020.1864
Elaphurus davidianus	NC_018358.1	
	JN632632.1	Hassanin, A., *et al.*, 2012. https://doi.org/10.1016/j.crvi.2011.11.002
Panolia eldii	NC_014701.1	
	JN632697.1	Hassanin, A., *et al.*, 2012. https://doi.org/10.1016/j.crvi.2011.11.002
Rusa alfredi	NC_020744.1	Hassanin, A., *et al.*, 2012. https://doi.org/10.1016/j.crvi.2011.11.002
Cervus canadensis	NC_050863.1	Kim, H.J., et al., 2020. https://doi.org/10.1080/23802359.2020.1780983
	NC_039923.1	Liu, H., et al., 2019. https://doi.org/10.1080/23802359.2019.1607588
	NC_014703.1	Li, Y., et al., 2014. https://doi.org/10.3109/19401736.2014.908373
Cervus elaphus	KT290948.1	Frank, K., et al., 2016. https://doi.org/10.1556/018.67.2016.2.2
	NC_007704.2	Wada, K., *et al.,* 2010. https://doi.org/10.1111/j.1740-0929.2010.00799.x
	AB245427.2	Wada, K., *et al.,* 2010. https://doi.org/10.1111/j.1740-0929.2010.00799.x
	KX449334.1	
	MF872247.1	Rey-Iglesia, A., *et al.,* 2017. https://doi.org/10.1002/ece3.3553
Rusa timorensis	NC_020745.1	Hassanin, A., *et al.*, 2012. https://doi.org/10.1016/j.crvi.2011.11.002
Rusa unicolor	NC_031835.1	Wu, X.*, et al.,* 2016. https://doi.org/10.1007/s12686-016-0547-6
	KY946815.1	Li, G., *et al.,* 2021. https://doi.org/1080/23802359.2021.1997118
Alces alces	JN632595.1	Hassanin, A., *et al.*, 2012. https://doi.org/10.1016/j.crvi.2011.11.002
	NC_020677.1	Hassanin, A., *et al.*, 2012. https://doi.org/10.1016/j.crvi.2011.11.002

Table S3. Substitution models as obtained from PartitionFinder, Related to STAR Methods. Partitions for the analyzed mitochondrial DNA used in this study in the Bayesian and divergence time estimation analyses.

	Partition no.	Best model	Length (bp)	Genomic features
**Protein coding genes**	1	GTR+I+G	4274	ND4L1, CYTB1, ATP8-1, ATP6-1, rrnL1, ND2-1, rrnS1, ND5-2, rrnS3, rrnS2, rrnL3, rrnL2
	2	HKY+I	3805	ND6-3, ND4L2, ND1-2, ND3-2, ND2-2, ATP6-2, ND5-3, CYTB2, COX2-2, COX3-2, COX1-2, ND4-2, ATP8-2
	3	HKY+G	2230	ATP8-3, ATP6-3, ND4L3, COX2-3, COX1-3, ND4-3, ND4-1, ND6-1
	4	SYM+I	1618	ND6-2, COX3-1, ND3-1, COX1-1, COX2-1, ND1-1
	5	GTR+G	2034	CYTB3, COX3-3, ND5-1, ND3-3, ND1-3, ND2-3
**Full mitogenomes**	**Partition no.**	**Best model**	**Length (bp)**	**Genomic features**
	1	GTR+I+G	4959	ATP8-1, trna_His, trna_Tyr, trna_Ala, trna_Phe, ATP8-2, trna_Val, trna_Gly, trna_Arg, trna_Pro, trna_Asp, trna_Ser2, trna_Trp, ATP6-2, trna_Thr, trna_Ile, ND2-3, ND5-3, rrnS3, rrnS2, rrnS1, rrnL3, rrnL2, trna-Gln, trna_Glu, rrnL1, trna_Lys
	2	HKY+I	4004	trna_Leu, trna_Ser, trna_Asn, trna-Met, ND4-3, ND6-3, ND5-1, ND3-1, CYTB3, ND4L1, ND2-1, ATP6-3, ND1-2, COX2-2, COX3-3, COX1-3
	3	SYM+I	1180	ND3-3, COX1-2, ND1-1, COX2-1
	4	GTR+G	2417	ND1-3, ND2-2, ND3-2, ND6-1, CYTB1, ATP6-1, COX3-1, ND5-2
	5	K80+I	911	trna_Cys, trna_Leu2, COX3-2, CYTB2, ND4L3
	6	HKY+G	1825	ATP8-3, ND4L2, COX2-3, COX1-1, ND4-2, ND4-1
	7	F81	170	ND6-2

Table S4. Log marginal likelihoods for the three independent runs under different clock models, related to STAR Methods. The marginal likelihoods were estimated in MrBayes using stepping-stone sampling. The model with the highest log-marginal likelihoods appears in bold font.

Protein coding genes		Full mitogenome	
Clock	log mle	Clock	log mle
**Strict**	**-38633.13**	**Strict**	**-48793.90**
Relaxed	-38737.78	Relaxed	-48882.64

Table S5. Combination of fossil and tree model priors to estimate divergence times with BEAUTi/BEAST v1.10.4 in fallow deer for our first dating approach, related to STAR Methods. A strict molecular clock and a mean clock rate value of 1.65 x 10^{-8} subs/site/year with a standard deviation of 0.01 (see Methods) were always used as priors. Lines with 0 fossil calibrations depict runs where only the substitution rate and/or tip ages were used for estimation. Likewise, lines with 1, 2 or 3 fossil calibrations depict the use of fossil constraints. When using just one fossil calibration, we used the fossil record estimate of 700 ka (± 50 ka) for the divergence between *D. mesopotamica* and *D. dama*. When using two fossil calibrations, we added the earliest possible occurrence of *M. giganteus* based on the fossil record (450 ka ± 100 ka). When using three fossil calibrations, we added the earliest *Rusa* fossil (3.4-2.6 Ma). Calibrations with a * were performed with the *Rusa* fossil node constraint established at the coalescence point of the *Dama-Megalocerus* clade with the *Cervus-Rusa-Elaphurus* clade. This was done to test the influence of divergence times provided by this fossil's age and position on age estimates.

Calibration priors	Tree prior	Tip ages used (age)	Estimated 95% HDP *Rusa-Cervus* split (Mean)	Estimated 95% HDP *M. giganteus-Dama* split (Mean)	Estimated 95% HDP *D. mesoptamica-D. dama* split (Mean)	Estimated 95% HDP crown *D. dama* split (Mean)
0 fossil calibration	Yule speciation	No	1.61–2.08 Ma (1.84 Ma)	2.19–2.84 Ma (2.51 Ma)	2.09–2.72 Ma (2.4 Ma)	119–195 ka (157 ka)
0 fossil calibration	Coalescent Bayesian skyline	No	1.63–2.14 Ma (1.89 Ma)	2.24–2.93 Ma (2.57 Ma)	2.11–2.79 Ma (2.45 Ma)	119–199 ka (160 ka)
0 fossil calibration	Coalescent Bayesian skyline	120 ka	1.62–2.11 Ma (1.87 Ma)	2.24–2.91 Ma (2.55 Ma)	2.12–2.77 Ma (2.43 Ma)	173–247 ka (208 ka)
1 fossil calibration	Yule speciation	No	1.4–1.72 Ma (1.56 Ma)	1.86–2.24 Ma (2.05 Ma)	1.74–2.09 Ma (1.91 Ma)	103–161 ka (130 ka)
1 fossil calibration	Coalescent Bayesian skyline	No	1.43–1.74 Ma (1.58 Ma)	1.87–2.25 Ma (2.06 Ma)	1.75–2.1 Ma (1.92 Ma)	102–161 ka (131 ka)
1 fossil calibration	Coalescent Bayesian skyline	120 ka	1.42–1.73 Ma (1.57 Ma)	1.87–2.26 Ma (2.07 Ma)	1.75–2.1 Ma (1.93 Ma)	158–213 ka (183 ka)
2 fossil calibration	Yule speciation	No	1.4–1.71 Ma (1.66 Ma)	1.86–2.24 Ma (2.05 Ma)	1.74–2.09 Ma (1.91 Ma)	101–161 ka (130 ka)
2 fossil calibration	Coalescent Bayesian skyline	No	1.44–1.75 Ma (1.58 Ma)	1.87–2.26 Ma (2.06 Ma)	1.74–2.1 Ma (1.92 Ma)	103–162 ka (131 ka)
2 fossil calibration	Coalescent Bayesian skyline	120 ka	1.42–1.73 Ma (1.57 Ma)	1.88–2.27 Ma (2.07 Ma)	1.76–2.11 Ma (1.93 Ma)	158–212 ka (183 ka)
3 fossil calibration	Yule speciation	No	2.21–2.65 Ma (2.39 Ma)	2.49–2.94 Ma (2.71 Ma)	2.27–2.67 Ma (2.46 Ma)	138–216 ka (175 ka)
3 fossil calibration	Coalescent Bayesian skyline	No	2.24–2.85 Ma (2.41 Ma)	2.5–2.95 Ma (2.72 Ma)	2.27–2.66 Ma (2.48 Ma)	140–217 ka (175 ka)
3 fossil calibration	Coalescent Bayesian skyline	120 ka	2.23–2.75 Ma (2.4 Ma)	2.5–2.96 Ma (2.72 Ma)	2.28–2.68 Ma (2.48 Ma)	188–261 ka (228 ka)

3 fossil calibration*	Yule speciation	No	1.57–1.85 Ma (1.71 Ma)	2.08–2.4 Ma (2.24 Ma)	1.92–2.23 Ma (2.08 Ma)	113–176 ka (142 ka)
3 fossil calibration*	Coalescent Bayesian skyline	No	1.59–1.86 Ma (1.73 Ma)	2.09–2.41 Ma (2.25 Ma)	1.94–2.24 Ma (2.08 Ma)	111–173 ka (143 ka)
3 fossil calibration*	Coalescent Bayesian skyline	120 ka	1.58–1.86 Ma (1.72 Ma)	2.1–2.42 Ma (2.25 Ma)	1.94–2.24 Ma (2.09 Ma)	167–225 ka (192 ka)

Table S6. Comparison of different calibration sets, Related to STAR Methods. Log marginal likelihoods for the comparison of independent runs under different models and data set calibration for age estimation in BEAST using stepping-stone sampling. Model evaluations were done based on the strict clock model as estimated by Mr. Bayes. The model with the highest log marginal likelihood is highlighted in bold font. CS = constant size, EG = exponential growth. Calibrations are depicted as: 1) Secondary *D. dama* 95% HDP interval obtained with the Yule tree prior and three fossil calibrations (mean= 175 ka; 95% CI: 138–220 ka), 2) Secondary *D. dama* 95% HDP interval obtained with the coalescent Bayesian skyline tree prior and no tip dating and three fossil calibrations (mean= 175 ka; 95% CI: 140–218 ka), 3) Secondary *D. dama* 95% HDP interval obtained with the coalescent Bayesian skyline tree prior, tip dating, and three fossil calibrations (mean= 228 ka; 95% CI: 188–262 ka).

Protein coding genes			
Clock	Tree model	Calibration set	log mle
Strict	CS	1	**-44196.7994**
Strict	CS	2	-44199.0098
Strict	CS	3	-44198.7604
Strict	EG	1	-44199.3667
Strict	EG	2	-44199.42
Strict	EG	3	-44199.3011
Full mitogenome			
Clock	Tree model	Calibration set	log mle
Strict	CS	1	-47752.9271
Strict	CS	2	-47753.024
Strict	CS	3	**-47752.9039**
Strict	EG	1	-47754.1538
Strict	EG	2	-47754.3356
Strict	EG	3	-47753.6411

Table S7. Pairwise log BayesFactors (BF) for all the BEAST models, Related to STAR Methods. Calculations were performed from the log marginal likelihoods stepping-stone sampling. Where the values are positive, the model in the row is better supported. CS = constant size, EG = exponential growth. Numbers depict the calibrations as described above.

Protein coding genes						
Log Bayes Factors						
	CS-1	CS-2	CS-3	EG-1	EG-2	EG-3
CS-1		2.2104	1.961	2.5673	2.6206	2.5017
CS-2	-2.2104		-0.2494	0.3569	0.4102	0.2913
CS-3	-1.961	0.2494		0.6063	0.6596	0.5407
EG-1	-2.5673	-0.3569	-0.6063		0.0533	-0.0656
EG-2	-2.6206	-0.4102	-0.6596	-0.0533		-0.1189
EG-3	-2.5017	-0.2913	-0.5407	0.0656	0.1189	
Full mitogenome						
Log Bayes Factors						
	CS-1	CS-2	CS-3	EG-1	EG-2	EG-3
CS-1		0.0969	-0.0232	1.2267	1.4085	0.714
CS-2	-0.0969		-0.1201	1.1298	1.3116	0.6171
CS-3	0.0232	0.1201		1.2499	1.4317	0.7372
EG-1	-1.2267	-1.1298	-1.2499		0.1818	-0.5127
EG-2	-1.4085	-1.3116	-1.4317	-0.1818		-0.6945
EG-3	-0.714	-0.6171	-0.7372	0.5127	0.6945	

References

Baker, K. H., Gray, W. I., Lister, A. M., et al. (2024). Ancient and modern DNA track temporal and spatial population dynamics in the European fallow deer since the Eemian interglacial. Scientific Reports 14(3015). doi: 10.1038/s41598-023-48112-6

Frank, K., Barta, E., Bana, N. A., et al. (2016). Complete mitochondrial genome sequence of a Hungarian Red deer (*Cervus elaphus hippelaphus*) from High-Throughput sequencing data and its phylogenetic position within the fasmuly Cervidae. Biologia Futura 67, 133 – 147. doi: 10.1556/018.67.2016.2.2

Hassanin, A., Delsuc, F., Ropiquet, A., et al. (2012). Pattern and timing of diversification of Cetartiodactyla (Mammalia, Laurasiatheria), as revealed by a comprehensive analysis of mitochondrial genomes. Comptes Rendus Biologies 335(1), 32–50.doi: 10.1016/j.crvi.2011.11.002

Kim, H. J., Hwang, J. Y., Park, K. J., et al. (2020). Complete mitochondrial genome of *Cervus canadensis* (Erxleben, 1777), as a model species of Chronic Wasting Disease (CWD). Mitochondrial DNA Part B 5(3), 2621–2623.doi: 10.1080/23802359.2020.1780983.

Li, Y., Ba, H., Yang, F. (2021). Complete mitochondrial genome of *Cervus elaphus songaricus* (Cetartiodactyla: Cervinae) and phylogenetic analysis with related species. Mitochondrial DNA Part A 27(1), 620–621. doi: 10.3109/19401736.2014.908373

Liu, H., Wang, T., He, J., Tu, J., Yang, X., Yang, F., Xing, X. (2019). The complete mitochondrial genome of *Cervus elaphus kansuensis* (Artiodactyla: Cervidae) and its phylogenetic analysis. Mitochondrial DNA Part B 4(1), 1720–1722. doi: 10.1080/23802359.2019.160758

Rey-Iglesia, A., Grandal-d`Anglade, A., Campos, P. F., Hansen, A. J. (2017). Mitochondrial DNA of pre-last glacial maximum red deer from NW Spain suggests a more complex phylogeographical history for the species. Ecology and Evolution 7(24), 10690–10700. doi: 10.1002/ece3.3553

Rey-Iglesia, A., Lister, A., Campos, P. F., et al. (2021). Exploring the phylogeography and population dynamics of the giant deer (*Megaloceros giganteus*) using Late Quaternary mitogenomes. Proceedings of the Royal Society B: Biological Sciences 288: 20201864. doi: 10.1098/rspb.2020.1864

Wada, K., Okumura, K., Nishibori, M., Kikkawa, Y., Yokohama, M. (2010). The complete mitochondrial genome of the domestic red deer (*Cervus elaphus*) of New Zealand and its phylogenetic position within the family Cervidae. Animal Science Journal 81(5), 551–557. doi: 10.1111/j.1740-0929.2010.00799

Wu, X., Qi, Y., Li, B., Yao, Y. (2016). The complete mitochondrial genome sequence of *Rusa unicolor dejeani* (Artiodactyla: Cervidae). Conservation Genetics Resources 8(3), 255–257. doi: 10.1007/s12686-016-0547-6
